# Evolution and Plasticity of the Transcriptome Under Temperature Fluctuations in the Fungal Plant Pathogen *Zymoseptoria tritici*

**DOI:** 10.3389/fmicb.2020.573829

**Published:** 2020-09-11

**Authors:** Arthur J. Jallet, Arnaud Le Rouzic, Anne Genissel

**Affiliations:** ^1^UMR BIOGER, Université Paris Saclay – INRAE – AgroParisTech, Thiverval-Grignon, France; ^2^UMR Évolution, Génomes, Comportement et Écologie, Université Paris-Saclay – CNRS – IRD, Gif-sur-Yvette, France

**Keywords:** adaptive evolution, fluctuation, experimental evolution, RNA-seq, temperature, gene expression, plasticity

## Abstract

Most species live in a variable environment in nature. Yet understanding the evolutionary processes underlying molecular adaptation to fluctuations remains a challenge. In this study we investigate the transcriptome of the fungal wheat pathogen *Zymoseptoria tritici* after experimental evolution under stable or fluctuating temperature, by comparing ancestral and evolved populations simultaneously. We found that temperature regimes could have a large and pervasive effect on the transcriptome evolution, with as much as 38% of the genes being differentially expressed between selection regimes. Although evolved lineages showed different changes of gene expression based on ancestral genotypes, we identified a set of genes responding specifically to fluctuation. We found that transcriptome evolution in fluctuating conditions was repeatable between parallel lineages initiated from the same genotype for about 60% of the differentially expressed genes. Further, we detected several hotspots of significantly differentially expressed genes in the genome, in regions known to be enriched in repetitive elements, including accessory chromosomes. Our findings also evidenced gene expression evolution toward a gain of robustness (loss of phenotypic plasticity) associated with the fluctuating regime, suggesting robustness is adaptive in changing environment. This work provides valuable insight into the role of transcriptional rewiring for rapid adaptation to abiotic changes in filamentous plant pathogens.

## Introduction

One remaining challenge in evolutionary genetics is to understand the role of regulatory variation in adaptation. In particular, in the context of rapid climate change with growing evidence that species experience more abiotic fluctuations (Fischer and Knutti, 2015), understanding the role of regulatory variants in adaptation to changing environments is increasingly attracting interest. Since early arguments in favor of regulatory variation explaining phenotypic differences between organisms (Britten and Davidson, 1971; King and Wilson, 1975), over the past two decades or so empirical studies have found abundant variation of gene expression within and between species, e.g., in Primates (Enard et al., 2002) including Humans (Rockman and Wray, 2002), in teleost fish (Oleksiak et al., 2002; Whitehead and Crawford, 2006), Drosophila (Wittkopp et al., 2004), and Saccharomyces (Fay et al., 2004). Heritable variation of gene expression within and between populations is widespread and much of which is thought to be adaptive (Brem and Kruglyak, 2002; Leder et al., 2015; Noormohammad et al., 2017; Glaser-Schmitt and Parsch, 2018).

Extensive body of work has shown that environmental changes cause rapid gene expression response [e.g., *Caenorhabditis elegans* (Li et al., 2006); *Escherichia coli* (Feugeas et al., 2016); *Drosophila melanogaster* (Zhou et al., 2012)]. Among all of the significant environmental factors, temperature is an important one. Many studies have reported large and pervasive effect of temperature on the transcriptome [e.g., using constant temperature stress (Podrabsky, 2004; Zhou et al., 2012; Chen et al., 2015; Jovic et al., 2017) or fluctuating temperature stress (Sørensen et al., 2016)].

Phenotypic plasticity is the ability for a genotype to produce different phenotypes in different environments (Pigliucci et al., 2006). Likewise a change in gene expression in response to temperature variation is considered as plasticity. Plasticity may play an important role for species adaptation to new environments by offering phenotypic variation prior to mutation accumulation. However our current understanding of the role of phenotypic plasticity in adaptation remains limited and it is still a matter of debate whether plasticity speeds up or impedes adaptation (Ghalambor et al., 2007; Levis and Pfennig, 2016; Fox et al., 2019). This question remains open as illustrated by recent work published in landmark papers either demonstrating adaptation to new environment by mean of plasticity – adaptive plasticity – (Ghalambor et al., 2015; Kenkel and Matz, 2016; Corl et al., 2018), or on the contrary supporting that plasticity does not facilitate adaptation (Ho and Zhang, 2018). In contrast to empirical studies insights from theoretical studies favor adaptive evolution of plasticity in fluctuating environment (Lande, 2009).

One powerful approach to study how gene expression and gene expression plasticity evolve in response to environmental variation is experimental evolution, which consists of laboratory-controlled evolution that lasts over several generations (Garland and Rose, 2009). The potential of experimental evolution in understanding adaptation to environmental fluctuations has been proven since the long term experimental evolutions in *Escherichia coli* under changing temperatures (Bennett et al., 1992; Leroi et al., 1994) or variable pH (Hughes et al., 2007). More recently, experimental evolution was also used to measure the effect of environmental variation on gene expression evolution [e.g., in *D. melanogaster* exposed to diet fluctuations (Huang and Agrawal, 2016; Zandveld et al., 2017); in *Saccharomyces cerevisiae* alternatively exposed to salt and oxidative stresses (Dhar et al., 2013); in *Caenorhabditis remanei* exposed to heat and oxidative stress (Sikkink et al., 2019)].

In contrast, there are fewer studies in genera deprived of historical model species, such as for filamentous fungi (Fisher and Lang, 2016). To fill this gap, we investigated the transcriptome evolution of the wheat fungal pathogen *Zymoseptoria tritici.* Toward this goal we analyzed the effect of temperature fluctuations on gene expression evolution and gene expression plasticity. Like other microorganisms, *Z. tritici* is an interesting model for experimental evolution due to its short generation time, small genome and the ease to maintain it in the laboratory. *Z. tritici* is a filamentous fungus, an ascomycete of the Mycosphaerellaceae family, and is the main causal agent of the Septoria Tritici Blotch disease of wheat (O’Driscoll et al., 2014; Fones and Gurr, 2015). *Z. tritici* is a haploid species that multiply asexually *in vitro* by budding (Steinberg, 2015). Nowadays this species becomes a good fungal model with growing interest to study its genome evolution since the publication of a complete reference genome (Goodwin et al., 2011). The genome of the reference strain has 21 chromosomes, including 13 gene-rich core chromosomes (CCs) and 8 accessory chromosomes (ACs) carrying less genes and more repetitive elements. While much attention has been paid toward genes underlying the ability of the pathogen to overcome the immune system of its host (through QTL linkage mapping (Meile et al., 2018; Stewart et al., 2018), or genome wide association studies (Hartmann et al., 2017; Zhong et al., 2017), we still have a poor understanding of the potential ability of *Z. tritici* to adapt to abiotic changes. Very few studies have found contrasted temperature sensitivity among natural population samples (Zhan and McDonald, 2011) or QTLs for thermal adaptation (Lendenmann et al., 2016). Transcriptome studies in the fungal pathogen *Z. tritici* are fairly recent and aimed for the most part at characterizing the waves of up- and down-regulated genes in association with symptom development inside the host plant (Kellner et al., 2014; Rudd et al., 2015; Palma-Guerrero et al., 2017).

Beside some evidence that there is a substantial amount of genetic variation among natural populations of *Z. tritici*, the contribution of regulatory variation to adaptation for this fungal species is virtually unknown. Here, we present the results of an experimental evolution to address several fundamental issues about fungal adaptation to thermal fluctuations: how does the transcriptome evolve in fluctuating temperature conditions, and to what extent is evolution repeatable between independent replicates and between genotypes? In particular, we test the assumption that phenotypic plasticity should evolve as an adaptation to a fluctuating environment.

We compared the transcriptome of evolved lineages maintained under stable or fluctuating temperature regimes, making use of 40 RNA samples obtained at two temperature treatments. We identified a pervasive effect of the selection regime on the gene expression level with a strong influence of the ancestral genotype. Results showed a few hotspots of significantly differentially expressed genes in the genome, in regions known to be enriched in repetitive elements including accessory chromosomes. Our results also showed a gain of gene expression robustness associated with the fluctuating regime as opposed to stable regime, suggesting robustness is adaptive in changing environment.

## Materials and Methods

### Biological Material

The two laboratory clones of *Z. tritici MGGP01* and *MGGP44* were collected on wheat in 2010 from the same population sample in the south of France at the location of Auzeville-Tolosane (43°53′ N – 1°48′W). For each clone, single colony was isolated and grown in the laboratory using Potato Dextrose Broth (PDB) medium at 17°C, 140 rpm and 70% humidity, for a few weeks. Strains were thus acclimated to our laboratory conditions prior to the experimental evolution. Strains were then multiplied for storage in PDB medium containing 30% glycerol at −80°C prior to the experimental evolution.

### Experimental Evolution Design

**FIGURE 1:**
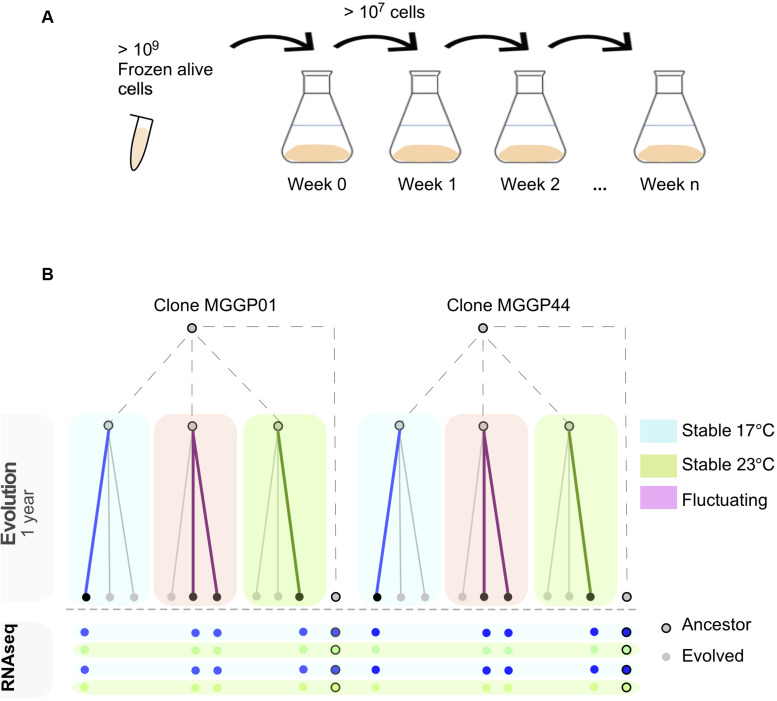
Experimental evolution approach using two lab clones of *Z. tritici*. *In vitro* weekly serial transfers occurred over 48 weeks in 500 ml liquid medium **(A).** After experimental evolution using three selection regimes (Stable at 17°C, Stable at 23°C and fluctuating between 17 and 23°C), 8 evolved lineages were RNA-sequenced in duplicate after 1 week growth at 17 or 23°C **(B)**.

Experimental evolution was conducted for two clonal strains of *Z. tritici* (*MGGP01* and *MGGP44*), by multiplying large asexual populations in the lab *in vitro*, using 500 mL of PDB at 140 rpm in a shaking incubator (Infors HT, Inc.). Every week 10^7^–10^9^ cells were transferred into fresh medium by pipetting 20 mL of cell suspension (Figure 1A). Three different selection regimes were used: two stable temperatures (17°C and 23°C) and a third condition with fluctuating temperature between 17 and 23°C rapidly every 52–64 h (Figure 1B). These two temperatures were non-stressful and allow sufficient cell growth by budding, as opposed to hyphal growth and mycelium formation induced by high temperature, nutrient-poor medium or oxidative stress (Francisco et al., 2019). In these laboratory conditions, we estimated that 1.5 mitotic division per day occurs for both genetic backgrounds, at 17 and 23°C. We thus estimate that 48 weeks of evolution correspond approximately to 500 generations. For each clone and abiotic condition three lineages were evolved. At the end of the experimental evolution after 48 weeks, large amounts of cell suspension were collected to provide liquid stocks in 30% glycerol at −80°C.

### RNA Isolation and Sequencing

RNA samples were isolated from the two ancestor clones and 8 evolved lineages (Figure 1B). As many samples as possible were sequenced under our budget constraints: 2 fluctuating, 1 stable at 17°C and 1 stable at 23°C for each ancestral genotype (*MGGP01* and *MGGP44*). The selection of the strains within each regime was random. This design allowed to analyze (1) the transcriptome evolution under fluctuation using stable lines as controls for high and low temperatures (2) gene expression plasticity evolution of each line when compared to the plasticity of the ancestor (see section “Evolution and Plasticity of Gene Expression”). The 10 samples (8 evolved lineages plus two ancestors) were grown in the same incubator for 1 week either at 17 or 23°C prior to collect the cell suspensions. Experiments were performed in duplicate at these two temperature conditions, in order to create two independent biological replicates. The full list of 40 RNA-seq samples is described in Additional File 1: Supplementary Table S1. Prior to RNA extraction of the 40 samples, 50 mL of snap frozen cell suspension were lyophilized for 3 days (LYOVAC^TM^ GT 2-E freeze dryer, Steris) and ground in liquid nitrogen to a fine powder with a mortar and pestle. Lyophilized tissue was homogenized in 1 mL of Trizol reagent (Invitrogen Inc.), and 200 μL of chloroform, vortexed, incubated at room temperature for 3 min and centrifuged at 12,000 g for 15 min at 4°C. Precipitation of RNA was done by transferring the aqueous phase to a new tube and adding 100% isopropanol. Samples were vigorously shaken and incubated at room temperature for 10 min. RNA pellets were obtained after centrifugation at 12,000 g for 10 min and washed with 1 mL of 70% ethanol. After centrifugation at 7,500 g for 5min, RNA was washed in 70% ethanol, air dried and re-suspended in RNase-free water and stored at −80°C. Sampling quality was checked using agarose gels, Qbit quantification (RNA HS assay kit, Molecular Probes Inc., United Kingdom) and Fragment Analyzer (Agilent, CA, United States). Libraries were created using TruSeq Stranded mRNA Sample Prep kit from Illumina by selecting fragments between 250 and 400 bp captured with poly-dT oligonucleotides (Integragen Inc., Evry, France). Paired-end (2 × 75 bp) sequencing was done with HiSeq4000 Illumina (Integragen Inc., Evry, France).

### Transcriptome Mapping and Quantification

We examined raw reads using FastQC (version 0.11.5)^1^, removed adaptors and performed quality-based trimming using Trimmomatic (version 0.32; Bolger et al., 2014) with the following options: PE -phred33 SLIDINGWINDOW:4:20 MINLEN:30 TOPHRED33. For each sample, mates of trimmed reads were mapped to the reference genome IPO-323^2^ using HISAT2 (version 2.0.4; Pertea et al., 2016). The default parameters were used except for the intron length, the number of multi mapping sites allowed per read and the stringency of mapping score, as follow: -min-intronlen 20 -max-intronlen 15,000 -k 3 –score-min L, 0, -0.4. We quantified the number of mapped reads using the annotation published by Grandaubert et al. (2015) without annotated sequence from transposable elements, using Stringtie with the following options: -p 16 -c 7 -m 200 -e –B –G (version 2.3; Pertea et al., 2016). Reads that mapped to the predicted 18S and 28S ribosomal units were all removed from the analysis. The final number of genes utilized for all analyses was 10950.

### Data Normalization

We performed read count normalization using a size factor calculated for each sample according to the RLE (Relative Log Expression) method implemented in the R package DESeq2 (Love et al., 2014). To identify expressed genes among different RNAseq samples coming from different batches of sequencing (strain IPO-323), we used Fragments Per Kilobase of transcript per Million mapped reads (FPKM) normalization.

### Coding Variant Calling From RNA-Sequencing

SNP calling for each RNA-sequencing sample was performed using GTAK GenomeAnalysisTK.jar with the following parameters: –T SplitNCigarReads –rf ReassignOneMappingQuality –RMQT 60 –U ALLOW_N_CIGAR_READS and GenomeAnalysisTK.jar –T HaplotypeCaller –dontUseSoftClippedBases –stand_call_conf 20 (DePristo et al., 2011). Custom R scripts were used to compare coding SNPs among samples.

### Natural Variation of Gene Expression Level Among Fungal Isolates

Gene expression levels were compared between our two ancestor clones and the reference strain IPO-323 (Dutch field strain isolated in 1984), using *in vitro* cultures RNA-seq data from the literature (using Yeast Malt Sucrose medium at 18°C (Kellner et al., 2014); using PDB medium at 18°C (Rudd et al., 2015); (fastq files *SRR1167717*, *SRR1167718*^3^; and fastq files ERR789217, ERR789218 kindly provided by J. Rudd). Raw fastq files from both studies were analyzed the same way as for our samples.

The following model was fit for each gene to compare the genotypes:

Y=ijμ+G+iεij,(DESeq2Model1)

Y*_*ij*_* is the expression level of the *i*th genotype (*i* = *IPO-323, MGGP01, MGGP44)* and the *j*th replicate (*j* = 1, 2, 3, 4), μ is the intercept, and *ε_*ij*_* is the random error. Significant effects were detected by comparing the full model to its null model without the genetic effect, using LRT and contrasts tests. Correction for multiple testing was performed using a FDR at 5% (Benjamini and Hochberg, 2005).

### Differential Gene Expression Analysis Among Evolved Lineages

To compare the gene expression levels among all evolved lineages the following DESeq2 model was fit for each gene:

Y=lskjμ+G+lR+sT+kGR+lsε(DESeq2Model2)lskj

Y*_*lskj*_* is the observed expression level of the *l*th genetic background (*l* = MGGP44, MGGP01), the *s*th selection regime (*s* = fluctuating, stable 17, stable 23), the *k*th assay temperature (k = 17°C, 23°C) and the *jth* replicate (*j* = 1,2), μ is the intercept and *ε* is the random error. The significance of each term was tested using a LRT by comparing the full model with a reduced model without the considered term. Correction for multiple testing was done using FDR at 5%.

To observe the relative contribution of genetic background, temperature and selection regimes effects, we also transformed the read counts with the DESeq2 *rlog* function to perform a Principal Component Analysis (PCA) with the DESeq2 *plotPCA* function. We computed the PC scores of each sample along the first four principal components. For each axis PC scores were then analyzed with the following ANOVA model using *lm* function in R:

PC=lskjμ+G+lR+sT+kε(ANOVAModel)lskj

PC*_*lskj*_* is the PC score of the *l*th genetic background (*l* = MGGP44, MGGP01), for the *s*th selection regime (*s* = fluctuating, stable 17, stable 23), at the *k*th assay temperature (k = 17°C, 23°C) and the *j*th replicate (*j* = 1, 2), μ is the intercept and *ε* is the random error.

### Evolution and Plasticity of Gene Expression

We analyzed the transcriptome evolution and the level of plasticity of gene expression for each ancestral genotype separately. Significant differences in the interaction term between lineages and temperature could not be tested with the DESeq2 Model (2), due to model overfitting. Here, the following model which includes the gene expression data from the ancestors was fit for each gene separately with the R package DESeq2:

Y=ijkμ+L+iT+jLT+ijε(DESeq2Model3)ij

Y*_*ijk*_* is the expression level of the *i*th lineage (*i* = *ancestor MGGP01, 12F, 13F, 1217, 1323*, or *ancestor MGGP44, 441F, 443F, 44117, 44323)*, at the *k*th temperature (*k* = 17°C, 23°C), and for the *j*th replicate (*j* = 1, 2), μ is the intercept, and *ε_*ikj*_* is the random error. LRT tests were used to compare the full model to reduced models, and FDR threshold at 5% was used for multiple testing correction.

To identify gene expression evolution due to fluctuation, significant results were classified based on the following rationale. Among genes that showed a significant lineage effect, we considered those with a significant contrast between evolved lineage and their ancestor. Genes exclusively evolving under fluctuation were pulled out when satisfying these two requirements: (1) significant contrasts between fluctuating and stable regimes (2) not significant contrasts between stable regime and ancestor.

To identify plastic genes, we considered genes with significant temperature effect with a fold change of read count greater than 2. To analyze the evolution of plasticity and compare plasticity between the two ancestors, fluctuating and stables lineages, we pulled genes using contrasts.

### Gene Ontology Enrichment Tests

To determine whether particular classes of gene function were overrepresented within the different sets of genes showing significant effects, we performed a Gene Ontology enrichment analysis for each set separately, using the R package GoSeq (Young et al., 2010). Note that 5690 genes are associated with unknown function in the current annotation of *Z. tritici*.

### Gene Co-expression Network Analysis

We searched for correlated patterns of expression among genes across the selection regimes. Only genes that displayed at least one significant contrast for the selection regimes from our DESeq2s *Model (3)* were included in this analysis (3174 and 3440 genes, for the genetic background *MGGP01* and *MGGP44*, respectively). Scale-free co-expression networks were built using a Weighted Gene Correlation Network Analysis with the R package WGCNA (weighted correlation network analysis) (Langfelder and Horvath, 2008). Using the log2 transformed read counts, the genes were clustered into modules according to their expression profile in response to the selection regimes and the assay temperatures. Modules were summarized by the first principal component of the gene expression data of the module (the eigengene), and the hub (most highly connected node of the module). All genes within modules were ranked using their correlation coefficient to the eigengene of the module.

### Statistical Analyses

All statistical tests were performed with R (version 3.3.1)^4^. In addition to the statistical models used for differential expression analyses, chi-square tests were used to compare the number of genes showing significant effects among chromosomes. Agreement testing between replicates was done using Cohen’s weighted kappa from “psych” R package. To do so we grouped raw read counts into 11 levels used as ordinal categories as follows: < 10 reads, between 10 and 25 reads, between 25 and 50 reads, between 50 and 100, between 100 and 250, between 250 and 500, between 500 and 1000, between 1000 and 2500, between 2500 and 10,000 and greater than 10,000.

Welch’s two samples *T*-tests were used to compare the length between up and down-regulated genes which expression level evolved after fluctuation. For the analysis of gene expression plasticity, chi-square tests with Holm’s correction for multiple testing were applied to compare changes of gene number among the different selection regimes. Following the WGCNA permutation tests (10,000 bootstraps) were done to test for the significance of the ranking position of candidate genes with functional annotation related to transcription regulation or signal transduction. Two bootstraps were done, using a sampling of 24 (genes involved in transcriptional regulation) or 39 (genes involved in signal transduction) randomly chosen genes among 4786 (total number of genes in the 8 selected modules revealed by WGCNA, Additional File 11: Supplementary Table S7).

## Results

Using two founder clones (hereafter labeled as MGGP01 and MGGP44), we performed a serial transfer experiment for 48 weeks, testing three abiotic conditions: two stable regimes (temperature of 17 or 23°C), and one fluctuating regime by alternating between these two temperatures every 2.5 days (Figure 1). At the end of the experiment, the transcriptome of 8 evolved lineages and ancestral clones was sequenced after 1 week multiplication at both low (17°C) and high (23°C) temperatures (see list of samples in Additional File 1: Supplementary Table S1).

### Mapping Quality and Normalization

The mapping rate on the genome from the reference strain IPO-323 (Goodwin et al., 2011), was greater than 83.5% for each sample. Average count of mapped reads was 35 million after relative log expression normalization, and similar distributions of normalized read count was observed (Additional File 2: Supplementary Figures S1, S2). Correlation coefficients between our biological replicates (Kendall τ) were on average 0.89, and agreement between those replicates was good, with Cohen’s weighted κ ranging from 0.86 to 0.97 (McIntyre, 2011), making the repeatability suitable for further analysis (Additional File 2: Supplementary Figure S3). Only 10% of the genes changed in expression by more than 2-fold between biological replicates. We tested our statistical models with and without this set of genes and we found very strong correlations of *P*-values. We thus included the full set of genes in our analyses presented here. Notably, in comparison to growth at 17°C, growth at 23°C resulted in a greater variation of gene expression across replicates (CV = 0.021 versus CV = 0.049, at 17 and 23°C, respectively).

### Genome-Wide Analysis of the Relative Abundance of Transcripts Between Acclimated Laboratory Strains

We checked the quality of our experiment by comparing our RNA-seq data with two previously published studies on the reference strain IPO-323 (Kellner et al., 2014; Rudd et al., 2015). For this purpose we used the transcriptomes from the two ancestor clones MGGP01 and MGGP44. In total, 61% of the genes were transcribed in all three genotypes (with 6663 common gene transcripts on the core chromosomes and 39 common gene transcripts on the accessory chromosomes among 10,950 genes (Table 1). Correlations between genotypes were examined using FPKM data (Additional File 3: Supplementary Figure S4). As expected, the highest correlation was found between the two ancestor clones coming from the same population (Pearson’s coefficient of 0.76). Comparisons of gene expression on CCs show congruent patterns between pairs of genotypes (Additional File 3: Supplementary Figure S5). Nevertheless, 57% (3768 out of 6663) of common transcripts were differentially expressed between genotypes (DESeq2 model 1) (Table 1). Some accessory chromosomes showed no transcriptional activity (ACs 16 and 21 for the genetic background MGGP01 and ACs 18 and 21 for MGGP44). Overall, ACs possessed a significantly lower proportion of expressed genes than CCs (*Chi*-2 test using a correction for the number of genes per chromosomes, *P* < 2.2 × 10^–16^). Among CCs chromosome 7 was singular. This chromosome contained significantly less gene transcripts than the other CCs, except for the smallest core chromosome 13 (pairwise *Chi-2* tests with a correction for the gene number, *P* < 0.025, except for CC7 vs. CC13, *P* = 0.057). A large fragment of 800 kb on the distal region of the left arm of chromosome 7 was without any transcriptional activity in all three genotypes (Additional File 3: Supplementary Figure S6). In conclusion, *in vitro* transcriptome between strains is similar for the identity of gene transcripts, but highly variable for the level of gene expression.

**TABLE 1:** Number and percentage of transcripts on core and accessory chromosomes for three fungal isolates (> 4 FPKM).

		Number of transcripts per genotype (%)			Common transcripts (%)	
Chromosome	Gene number	*IPO-323*	*MGGP01*	*MGGP44*	Number	Significant genotype effect
1	1986	1515 (76)	1589 (80)	1604 (81)	1419 (71)	772 (54)
2	1146	845 (74)	894 (78)	906 (79)	781 (68)	454 (58)
3	1073	770 (72)	834 (78)	840 (78)	715 (66)	398 (55)
4	824	605 (73)	652 (79)	667 (81)	554 (67)	324 (58)
5	782	559 (71)	590 (75)	591 (76)	505 (64)	271 (53)
6	692	489 (71)	518 (75)	516 (75)	446 (64)	247 (55)
7	743	385 (52)	402 (54)	412 (55)	347 (46)	190 (55)
8	694	518 (75)	538 (78)	527 (76)	466 (67)	273 (58)
9	604	412 (68)	409 (68)	427 (71)	357 (59)	211 (59)
10	516	359 (70)	375 (73)	376 (73)	325 (63)	189 (58)
11	488	332 (68)	339 (70)	352 (72)	298 (61)	172 (58)
12	414	287 (69)	308 (74)	307 (74)	266 (64)	157 (59)
13	334	210 (63)	230 (69)	224 (67)	184 (55)	110 (60)
Total core	10296	7286 (71)	7678 (75)	7749 (75)	6663 (65)	3768 (57)
14	114	32 (28)	22 (19)	20 (18)	17 (15)	14 (82)
15	86	14 (16)	25 (29)	8 (9)	6 (7)	4 (66)
16	88	17 (19)	0	4 (5)	0	0
17	78	5 (6)	22 (28)	3 (4)	2 (2)	2 (100)
18	64	0	18 (28)	0	0	0
19	87	18 (21)	14 (16)	11 (13)	7 (8)	6 (86)
20	79	12 (15)	25 (32)	11 (14)	7 (9)	4 (57)
21	58	24 (41)	0	0	0	0
Total accessory	654	122 (18)	126 (19)	57 (9)	39 (6)	30 (77)

*Chromosome and gene numbers are from the reference isolate IPO-323; expression data for the reference strain IPO-323 were taken from Kellner et al. (2014) and Rudd et al. (2015).*

### Contrasted Transcriptome Evolution Between Genetic Backgrounds

**FIGURE 2:**
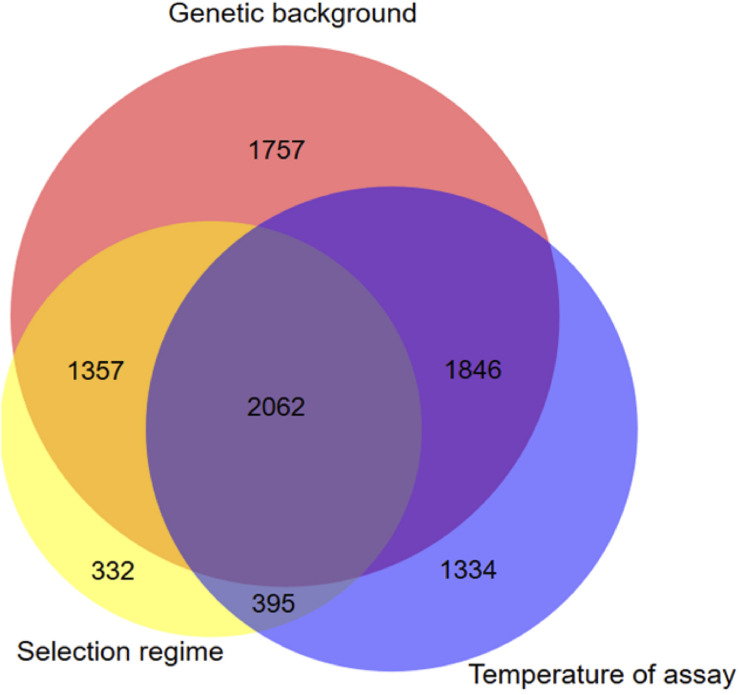
Venn diagram displaying unique and shared DEGs due to selection regimes, temperature and genetic background. Genes with significant selection regime effect were obtained using the statistical model DESeq2 model (*2*) which includes all evolved sequenced lineages.

To evaluate whether there was any differences of gene expression among the three selection regimes, we first compared the transcriptomes without the ancestor data. The genetic background and the assay temperature were the two main factors contributing to the total variance among evolved lineages (Table 2) (Principal Component Analysis, with 51% (98% of PC1) and 11.3% (66% of PC2 and 20% of PC3), respectively). The contribution of the selection regime (stable 17°C, stable 23°C, or fluctuating) was smaller with 6% of total variance (30% of PC3 and 66% of PC4) but significant. We then used our DESeq2 model (*2*) to identify the set of genes differentially expressed between both genotypes, between selection regimes, and between temperature treatments. We found 7022 genes that showed significant differences of expression between the two genetic backgrounds, 4146 genes that differed across selection regimes and 5637 genes that differed due to the assay temperature (Figure 2). In addition, we detected 1728 differently expressed genes for the interaction term between genetic backgrounds and selection regimes. More specifically, many of those genes showed opposite direction of differential expression between the two genetic backgrounds (Additional File 4: Supplementary Figure S7). In sum, the expression level of a large number of genes evolved differently in both genetic backgrounds. As a consequence, to gain full insight in gene expression changes in response to the selection regime, we considered the two ancestral genetic backgrounds separately in further analyses.

**TABLE 2:** Total percent variation in gene expression explained from DeSeq2 Model (*2*).

Principal components	PC1 (52%)	PC2 (15%)	PC3 (7%)	PC4 (6%)
Genetic background	***	NS	NS	NS
	(98%)			
Assay temperature	NS	***	**	NS
		(66%)	(20%)	
Selection regime	NS	NS	**	***
			(30%)	(66%)
*Residuals*	*(1%)*	*(34%)*	*(50%)*	*(32%)*

Contribution (percent of variance) of the genetic background; assay temperature and selection regime to the first four Principal Components (PC1, PC2, PC3, PC4), significance is indicated by NS (non-significant), **(P < 0.01); ***(P < 0.001).

### Evolution of Gene Expression During Adaptation to Fluctuation

We investigated the evolution of gene expression by including the data from the ancestor (DESeq2 model *3*). Evolution of gene expression in response to selection was inferred from significant differences between ancestral and evolved transcriptomes.

**FIGURE 3:**
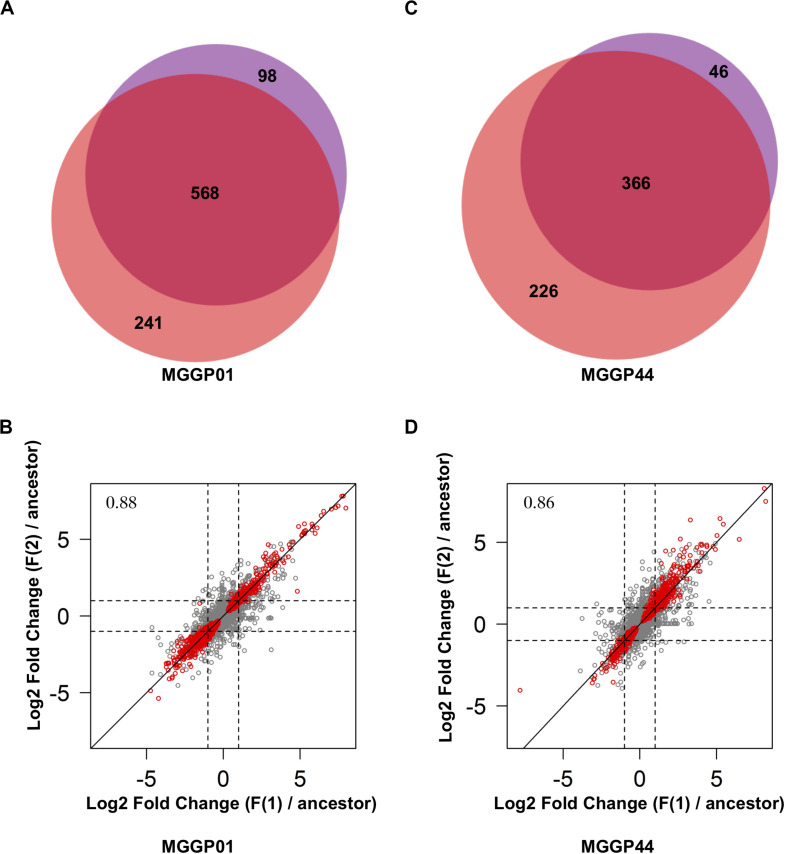
Comparison of DEGs between the two replicates of evolution under fluctuation. Venn diagrams showing the number of genes evolving in response to fluctuation that are common between replicates for MGGP01 **(A)** and MGGP44 **(C)**. Correlation of Log2 Fold change (evolved lineage against ancestor) between replicates under fluctuation (F1 and F2), for MGGP01 **(B)** and MGGP44 **(D)**, respectively, Pearson’s correlation coefficient on whole data: ρ = 0.88 and 0.86, respectively. Red points: significant genes after FDR correction at 5%.

Thirty two percent of the genes (3420 and 3659 genes for MGGP01 and MGGP44, respectively) were differentially expressed across selection regimes (Table 3). The assay temperature also affected the level of gene expression, for up to 39% of the transcriptome. The distribution of the fold-change of gene expression for all genes showing a significant effect is given in the Additional File 5: Supplementary Figure S8. The number of genes affected by fluctuation was approximately the same in both genetic backgrounds. Nonetheless, the number of up- and down-regulated genes was asymmetric between the two genetic backgrounds, with a majority of evolution toward down-regulation for MGGP01, and an opposite pattern for MGGP44 (Table 3, Additional File 5: Supplementary Figures S8, S9). As the transcriptome was sequenced in two independent fluctuating lines for each background, we were able to measure the amount of parallel evolution in our experimental setting. Significant change in expression compared to the ancestor was highly correlated in parallel lineages for each genetic background with a large proportion (nearly 60%) of common genes between the two independently evolved lines (568 out of 907 Differentially Expressed Genes (DEGs) for MGGP01, and 366 out of 638 DEGs for MGGP44 when considering genes with a fold change greater than two) (Figure 3). We focused our attention on this set of genes, assuming that parallel evolutionary change could be the consequence of adaptive evolution in fluctuating environments. When comparing the two genetic backgrounds we detected an overlap of 70 DEGs due to fluctuation, with 45 genes evolving in the same direction. Notably, the gene length between up- and down-regulated genes in fluctuating conditions was biased toward a smaller size for down-regulated genes (*Welch t-*tests, *P* = 3.8 × 10^–9^ and *P* = 0.003, for MGGP01 and MGGP44, respectively).

**TABLE 3:** Number of DEGs associated with the selection regime and the assay temperature.

	Background *MGGP01*	Background *MGGP44*
Selection regime (overall effect)	3420	3659
*Stable 17*°*C*	244 (3.1)	588 (2.9)
*Stable 23*°*C*	436 (3.1)	532 (2.8)
*Fluctuating*	666 (2.9)	412 (2.8)
*Fluctuating*	809 (2.7)	592 (2.8)
Temperature	3601	4333
Regime-by-Temperature	414	851

*The number of genes showing significant differences between ancestral and evolved lineages with a fold-changed > 2 is considered (stable at 17°C, stable at 23°C and fluctuating between 17°C and 23°C). Each genetic background (MGGP01, MGGP44) was analyzed separately. Median fold change among significant contrasts is indicated in parentheses.*

**FIGURE 4:**
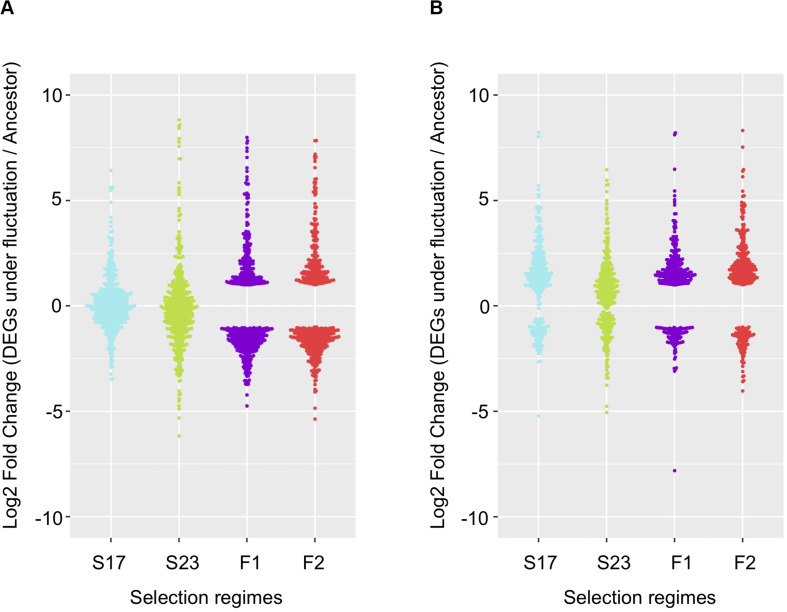
Repeatability of evolution of gene expression under fluctuating temperature regime. 568 repeated DEGs for the background MGGP01 **(A)**; 366 repeated DEGs for the background MGGP44 **(B)**; selection regimes are annotated as follow: Stable regimes (S17 and S23); fluctuating regimes (experimental evolution replicates F1 and F2). Log2 Fold changes are relative to the ancestral gene expression level.

**FIGURE 5:**
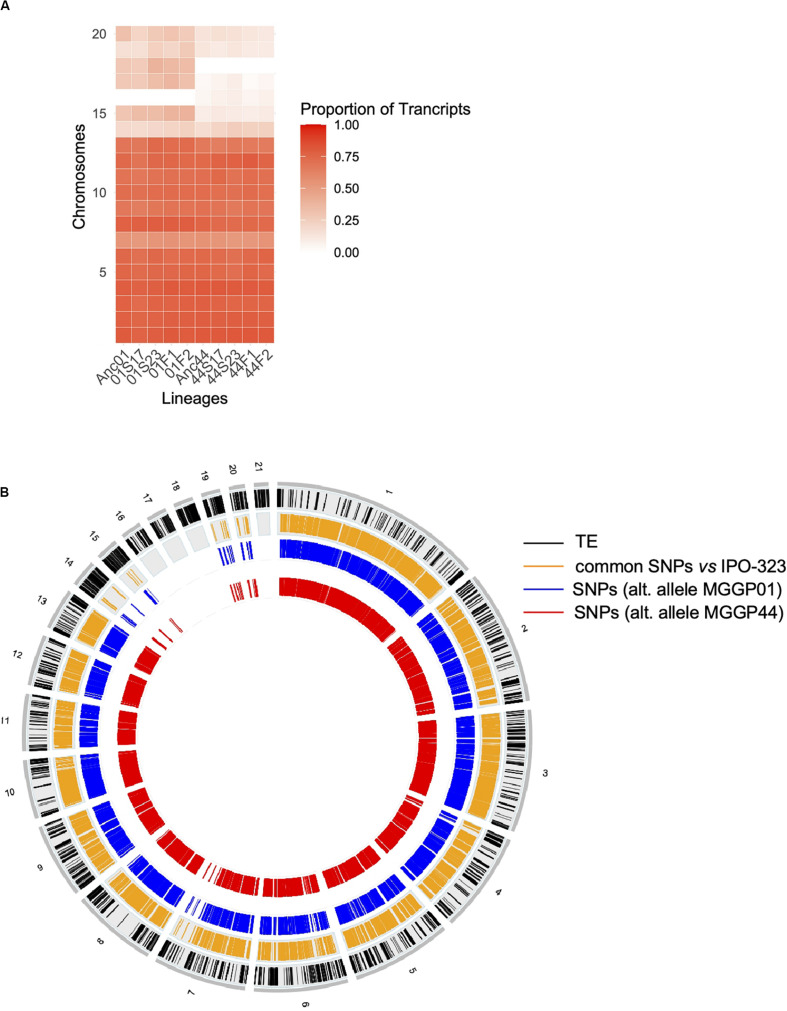
Evidence for genetic variation among ancestors. Proportion of transcribed genes per chromosome among lineages compared to the number of annotated genes in the reference genome IPO-323 **(A)**. Ancestor MGGP01 (Anc01), lineage stable 17°C MGGP01 (01S17), lineage stable 23°C MGGP01 (01S23), lineage fluctuating temperature F1 and F2 (01F1 and 01F2, respectively), ancestor MGGP44 (Anc44), lineage stable 17°C MGGP44 (44S17), lineage stable 23°C MGGP44 (44S23), lineage fluctuating temperature F1 and F2 (44F1 and 44F2, respectively); SNPs in transcripts between ancestors and compared to IPO-323 **(B)**. Data are plotted in 50kb windows, using the Bioconductor OmicCircos R package. From outer to inner ring: TE positions in IPO-323 reference strain from Rudd et al. (2015) (black); SNPs between the MGGP strains and IPO-323 (orange); SNPs between MGGP01 and MGGP44: MGGP01 different than the reference (blue); MGGP44 different than the reference (red).

Among genes significant for the fluctuating regime, a large number were also significant for the stable regimes, to a greater extent for the background MGGP44 (with 90 and 61% of genes in common between fluctuating and stable regimes at 17°C and 23°C, respectively, versus 21 and 41%, for the background MGGP01) (Figure 4). While 20 transcripts were specific to the fluctuation regime for the genetic background MGGP44, 291 genes specifically evolved under fluctuations for the background MGGP01 (gene lists are given in Additional File 6: Supplementary Table S2). This strong contrast between the two genetic backgrounds illustrates the genetic diversity of this fungal species, contributing to the complex genetic basis of adaptation. In this study there is evidence for high level of genetic variation between two ancestors (Figure 5). First, we noticed a presence-absence polymorphism of the mini chromosomes (Figure 5A). While AC 21 seemed to be missing for both strains, AC 16 had no transcripts for MGGP01, and AC 18 had no transcripts for MGGP44. In addition, we detected many SNPs in common transcripts between the two ancestors, with 62112 SNPs between MGGP01 and MGGP44, and 22384 SNPs between IPO-323 and the two ancestors (Figure 5B).

### Gene Ontology Tests

Gene Ontology (GO) term enrichment tests identified different functional terms between the two genetic backgrounds. First, when considering the 568 genes having evolved in the fluctuation regime for the wild-derived background MGGP01, we did not find any evidence for GO term enrichment. However, when considering only the 284 genes that evolved specifically under thermal fluctuations, we detected functional categories related to *Cell cycle control, cell division and chromosome partitioning* (*P* = 0.0019) and *Cytoskeleton* (*P* = 0.027). These functional categories include proteins known to be essential components of the cytoskeletal mitotic apparatus and essential regulators of cell cycle progression, such as the gene ID 59920, ortholog of the conserved regulator of mitosis Wee1, which is known to control the timing of entry into mitosis in yeast (Kellogg, 2003; Additional File 6: Supplementary Table S2). For the lineages from the background MGGP44, when considering all significant genes for the fluctuating regime (which also appeared significant for the stable regimes), the GO term *defense mechanisms* was found (9 genes, *P* = 0.013, with 6 genes up-regulated and 3 genes down-regulated, see Additional File 6: Supplementary Table S2).

### Genome Wide Distribution of DEGs

**FIGURE 6:**
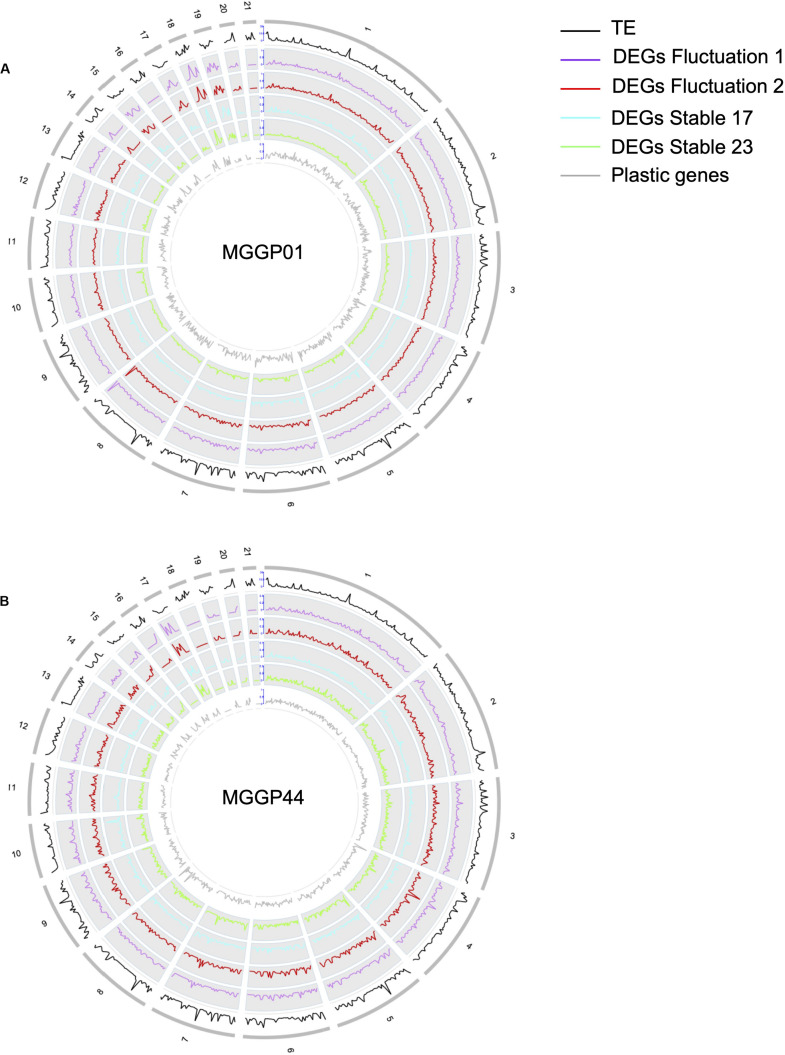
Whole genome distribution of regulatory effects. Gene density profiles for MGGP01 **(A)** and MGGP44 **(B)**, respectively. Transposable element density (IPO-323), DEGs density for each selection regime, and DEGs due to the assay temperature are given within 50 kb windows along each chromosome.

We further examined the genomic distribution of differentially expressed genes due to the selection regimes. First, we contrasted the number of differentially expressed genes between CCs and ACs. For the lineages derived from the background MGGP01 only, the proportion of DEGs normalized by the total number of genes per chromosome was higher on ACs than on CCs (Additional File 7: Supplementary Table S3). Next, we surveyed the density of DEGs along each chromosome using a non-overlapping window approach (Figure 6). The distribution of the genes with significant change of expression during adaptation to stable and fluctuating regimes was not uniform, as we found several regions where the gene density was high. Unexpectedly, these hotspots with a higher density of DEGs –normalized by the number of known annotated genes– associated with thermal adaptation were sometimes located near the tips of chromosomes: 2, 6, 7, 8, and 13 for the CCs or across the ACs with mainly chromosomes 15, 17, 18, and 19. In contrast, plastic genes that showed a significant temperature effect using the same model (DESeq model *3*) were heterogeneously distributed along the genome with no clear evidence of hotspots (Figure 6). The enrichment of accessory chromosomes in genes which expression changed during experimental evolution suggests that these chromosomes could be involved in adaptation to abiotic environment. Overall, about 40 genes had their expression turned off after experimental evolution (only 2 genes on ACs). None of these genes were located in hotspot regions, thus excluding the hypothesis that the hotspot regions could correspond to deletion events. As a matter of fact, 80% of the genes located in hotspots were up-regulated compared to the ancestral gene expression level. These hotspots often collocate with regions known to be enriched in transposable elements and are often near subtelomeric regions of core chromosomes (Schotanus et al., 2015). This result suggests that various evolutionary scenarios, including a rapid turn-over of gene regulation in these regions where transposition during mitosis, could impact gene expression. Such hypotheses remain difficult to test without further experimental evidence.

### Gene Network Rewiring

We performed a weighted correlation network analysis to search for clusters of co-regulated genes (details in “Materials and Methods” section). A total of 16 and 15 co-expression modules were detected, respectively, for MGGP01 and MGGP44 lineages (Additional File 8: Supplementary Tables S4, S5). We retained 8 of the largest co-expression modules that include most of the genes which were significant for the fluctuating regime from our model (*3*) (63 and 92%, for MGGP01 and MGGP44, respectively) (Additional File 9: Supplementary Figure S10). The goal of this approach was to identify hub genes within co-expression modules, defined as the closest gene from the module average (Additional File 10: Supplementary Table S6). Several molecular functions were involved, including histone modification and phospholipase activity. Interestingly, among the 8 modules, we found 23 genes involved in transcriptional regulation and chromatin remodeling (mainly transcription factors) and 39 genes involved in signal transduction, including 11 genes with a kinase activity (Additional File 11: Supplementary Table S7). However, the ranking position of these genes within modules was not significantly biased toward the most connected genes (Wilcoxon tests with empirical permutation testing, *P* = 0.98 and *P* = 0.99, for transcription factors and signal transduction genes, respectively). When considering the relationship between the physical (genomic) distance between genes within modules with coexpression, no large *cis*- or *trans-*regulated clusters of genes were detected. Co-expressed transcripts within modules were rather scattered across the whole genome (Additional File 9: Supplementary Figure S11).

### Evolution of Gene Expression Plasticity

The effect of the assay temperature in our laboratory growth conditions – the gene expression difference between 17°C and 23°C – was large and pervasive across the genome (DEseq Model *3*, see Table 3). In order to make sure that differences in gene expression between temperatures could be attributed to plasticity, we verified that the genetic composition of the populations was identical at both temperatures for the genes influenced by temperature. Indeed, in a polymorphic population, where several clones could be competing at the end of the experimental evolution, we can assume that there is a confounded effect between clone competition at the two temperatures and actual gene expression plasticity. In other words, if the clone fitness varies between the two temperatures, different gene expression level could be due to changes in genotype frequency rather than single genotype differential expression. We observed new mutations in transcribed regions from the RNA sequences and measured mutation frequencies at both temperatures. We found no evidence for a change in haplotype frequency between samples (Additional File 12: Supplementary Table S8). Based on these results, we made the assumption that changes of gene expression were associated with phenotypic plasticity.

**FIGURE 7:**
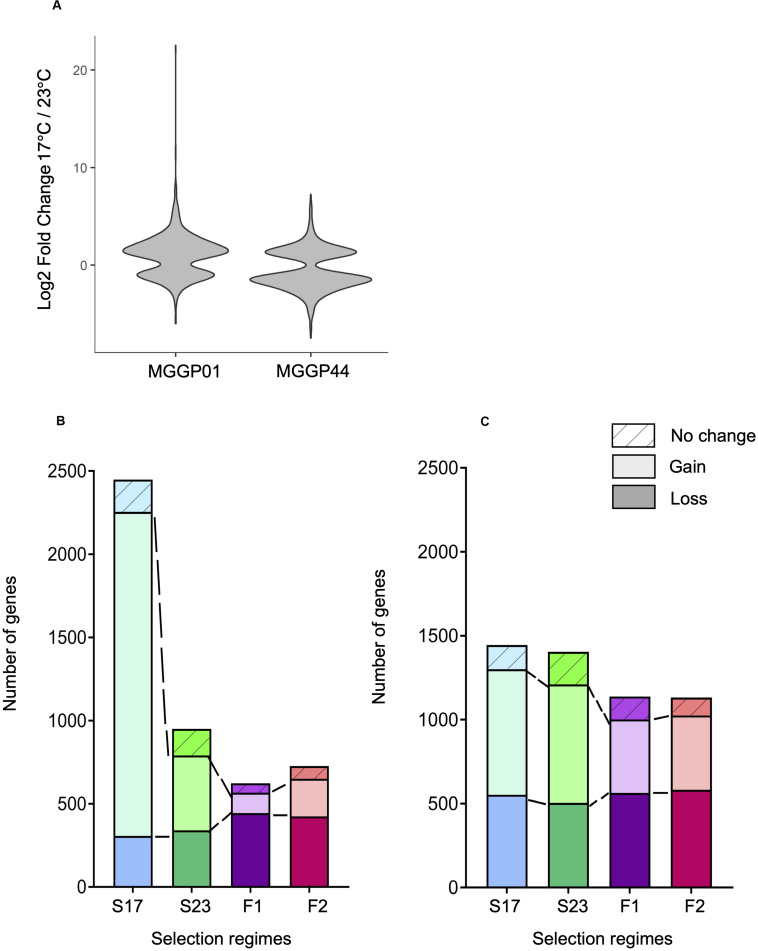
Evolution of plasticity for each selection regime. Fold change of gene expression between the two temperature conditions for the ancestors MGGP01 and MGGP44 **(A)**. Stacked bar charts describing the number of DEGs due to the assay temperature evolving toward a reduced plasticity (bottom), evolving toward a gain of plasticity (middle bar), and not evolving (top bar), for MGGP01 **(B)** and MGGP44 **(C)**.

The proportion of plastic genes between the two ancestors was significantly different. For a total of 7804 plastic genes, we report 653 versus 1348 plastic genes for MGGP01 and MGGP44, respectively (*P* = 2.2 × 10^–16^) (Figure 7). When considering genes with a raw fold change greater than two, we detected 501 versus 699 plastic genes for MGGP01 and MGGP44, respectively (*P* = 4.4 × 10^–8^). Among those plastic genes, 92 were common between the two ancestors. Interestingly, the distribution of the expression fold-change between the two temperatures was biased toward up-regulated genes at 17°C for MGGP01. Opposite result was found for MGGP44, with more up-regulated genes at 23°C (Additional File 13: Supplementary Table S9).

We identified 3.8 and 7.8% of the transcriptome displaying a significant Temperature-by-Regime interaction in at least one of the three selection regime, for wild-derived background MGGP01 and MGGP44, respectively. These genes were thus associated with an evolutionary change in phenotypic plasticity, which was further classified as a gain or loss of plasticity in comparison with the ancestral plasticity. In both genetic backgrounds, the fluctuating lines acquired less plastic genes than stable lines (Chi-square tests with Holm’s correction for multiple comparisons, all *P* < 2.10^–7^), with a significant loss of plastic genes when compare to stable lines in the background MGGP01 (Chi-square tests with Holm’s correction for multiple comparisons, all *P* < 2.10^–3^) (Figure 7). These findings suggest that in our laboratory conditions, adaptation to thermal fluctuations is not associated with the evolution of enhanced plasticity of gene expression. More surprisingly perhaps, adaptation to fluctuations tends to be associated with the loss of plasticity for a substantial number of genes that were plastic in the ancestor, suggesting that plasticity could even be maladaptive in a fluctuating environment.

## Discussion

### Intraspecific Variation of Gene Expression

By comparing three genotypes of *Z. tritici* we found pervasive gene expression variation most likely due to the contribution of numerous variants (small or large scale mutations) including variation of histone modification. Substantial amount of standing genetic variation is present in populations of this species (nucleotide diversity pi = 0.01) (Hartmann et al., 2018). Although transcriptomic comparisons are often made using a limited number of strains, there is accumulating evidence for a considerable variation of gene expression within and between populations in *Z. tritici* (Palma-Guerrero et al., 2017; Haueisen et al., 2019). In this study we evidenced that 57% of the transcriptome varies among genotypes. This level of variation leaves room for adaptation and is comparable to the amount of natural variation already reported within eukaryotic species, mostly using eQTL mapping (e.g., Schadt et al., 2003; Huang et al., 2015; Leder et al., 2015; Zan et al., 2016; Osada et al., 2017). Interestingly, we also found a large silenced region on chromosome 7 in all three wild genotypes. This region was previously identified for the strain IPO-323 by Kellner et al. (2014); Rudd et al. (2015), and Schotanus et al. (2015). This region is known to carry histone H3K27me3 modifications that mediate transcriptional silencing, also present on the ACs. This chromosomal fragment most likely originated from a fusion of a whole accessory chromosome onto the original chromosome 7 (Schotanus et al., 2015), and is in fact conserved among the three wild genotypes compared in this study. Likewise, Haueisen et al., 2019 recently confirmed the conservation of this silenced region among several other wild isolates.

### The Effect of Fluctuating Selection on the Transcriptome

Evolutionary change in gene expression was pervasive, with as much as 38% of *Z. tritici* transcripts displaying different expression levels among evolved lineages. In our experimental conditions evolved lineages had on average several hundreds of genes which expression level changed by at least a factor 2. Transcriptome evolution in fluctuating conditions was shown to be repeatable between parallel lineages initiated from the same clone. However, our results clearly show that evolution was not parallel when using different genetic backgrounds, with little overlap between differentially expressed genes. Interestingly, different functional categories were over-represented between the two genetic backgrounds, suggesting different evolutionary routes can be explored to respond abiotic changes. In contrast to MGGP01, the background MGGP44 showed a very few genes responding solely to the fluctuating regime. Beside the fact our strains were acclimated for several weeks prior to the experiment, we cannot exclude that many of those genes indeed evolved in response to the laboratory selection. Whether the ancestor MGGP44 was already adapted to fluctuating environment (e.g., through its initial large number of plastic genes), or that adaptation to fluctuations may not be based on a different set of genes than adaptation to stable conditions, remains to be determined. To sum up these results illustrate that this fungal species can evolve fast by means of gene expression variation operating on many functional categories, but the evolutionary outcome is different between genetic backgrounds.

### Genome-Wide Distribution of Regulatory Changes

The genome-wide distribution of differentially expressed genes due to selection suggests that a few genomic regions are more prone to rapid evolution. Compartmentalization of filamentous fungal genomes is a well-known feature, characterized by the presence of regions of distinct evolutionary rates (Dong et al., 2015; Frantzeskakis et al., 2019). In our experiment, regions near subtelomeres on a few core chromosomes and some accessory chromosomes were more prone to rapid evolution of gene expression. This result has important implications for understanding the evolutionary potential of filamentous fungi. Accessory chromosomes are often qualified as a cradle for adaptive evolution, due to their high mutation rates and structural rearrangements (Croll and McDonald, 2012). We provide here the first experimental evidence of rapid evolution of gene expression in this genome “accessory” compartment. However the molecular causes of gene expression changes remained to be understood. The regions enriched in genes showing significant effect of the selection regimes (tips of CCs and large portion of ACs), tend to contain more transposable elements than the genome average (Schotanus et al., 2015). The role of transposable elements in regulating gene expression evolution in Eukaryotes has been well documented (see Schrader and Schmitz, 2019 for recent review). These results suggest that further investigations are needed to identify the causal factors –TE themselves, epigenetic regulation, or SNPs/indels– that are associated with the evolution of gene expression level.

### Genome Stability During Asexual Multiplication

Loss of accessory chromosome has been recently reported for 4-week *in vitro* grown cells in yeast-malt-sucrose medium in *Z. tritici* (Möller et al., 2018). In our study we found no evidence for a loss of accessory chromosomes (among genes significant for selection the regimes, two were turned off on accessory chromosomes). However, we cannot exclude the hypothesis that some of the cells in evolved lineages lost accessory chromosomes, thus falsely leading to a down-regulated effect on gene expression. Likewise, up-regulation of gene expression could obscure a loss of ACs, and TE mobility could influence the genome structure and falsely lead to an increase of gene expression that is in fact associated with copy number variation.

### Fighting Changes by Staying Stable

We observed significant differences of plasticity between both ancestral genotypes, highlighting genetic variation for transcription plasticity. Genotype-by-environment interaction for gene expression is common in many organisms (e.g., yeast (Landry et al., 2006; Smith and Kruglyak, 2008; Li et al., 2010), *Arabidopsis* genus (He et al., 2016). Plasticity is often thought to be an adaptive response to environmental heterogeneity, that is likely to be lost in stable environments where it is likely to be unnecessary (Lande, 2009). In this context, our observation that gene expression plasticity tends to be lost in all lineages exposed to fluctuating selection regimes appears at odds with theory. This result indicates that in our experimental conditions robustness of gene expression is favored under environmental fluctuations. The evolutionary consequences of robustness are important. Robustness can be adaptive and lead to the accumulation of cryptic genetic mutations that can increase the evolvability of the species facing new environments (Le Rouzic and Carlborg, 2008; Cuypers et al., 2017; Payne and Wagner, 2019). Several theoretical approaches have explained a gain of robustness, which is dependent on the rate of fluctuation and the strength of selection (Siegal and Bergman, 2002; Le Rouzic et al., 2013). Full understanding of the molecular basis of robustness remains largely unexplored, with a clear lack of empirical support. Robustness could rely on functional redundancy arising from gene duplications, or alternatively on specific network topological features. Unfortunately, functional annotation of the genome of *Z. tritici* is not advanced enough yet to gain full insights into these questions. A recent empirical study in *Arabidopsis thaliana* did not support the above hypothesis and demonstrated that a single gene, rather than gene network connectivity or functional redundancy, was associated to trait robustness (Lachowiec et al., 2018). In contrast, a former study evidenced robustness by means of functional redundancy in yeast (Li et al., 2010).

## Conclusion

Our findings demonstrate substantial evolution of gene expression under fluctuating temperature with striking differences between genetic backgrounds. Notably, we identified a set of genes involved in gene expression, chromatin regulation and signal transduction genes. The ecological relevance of those genes in the adaptation to temperature variation remains to be elucidated. In addition our results suggest an important role of gene expression evolution on the accessory chromosomes, which has not been reported before for this species. Last, we found that under fluctuating temperature the transcriptome evolved toward a loss of plasticity. These findings suggest more work is needed to further characterize the role of gene expression plasticity in adaptation.

## Data Availability Statement

The datasets for this study can be found in the NCBI repository (SAMN13915588–SAMN13915627, https://www.ncbi.nlm.nih.gov/sra, .bam format).

## Author Contributions

AL and AG conceived the experiments. AG performed the experimental evolution. AJ conducted all the bioinformatics analysis. AJ, AL, and AG performed the statistical analyses, interpretation of the data, and writing.

## Conflict of Interest

The authors declare that the research was conducted in the absence of any commercial or financial relationships that could be construed as a potential conflict of interest.

## Footnotes

1 https://www.bioinformatics.babraham.ac.uk/projects/fastqc/

2 http://genome.jgi.doe.gov/Mycgr3/Mycgr3.home.html

3 https://www.ncbi.nlm.nih.gov/sra?term=SAMN02640204

4 https://www.r-project.org

## Supplementary Material

FILE S1 Supplementary Table S1. Full list of RNA samples from the experimental evolution used for the differential gene expression analysis (Pdf 94KB).

FILE S2 Supplementary Figures S1–S3. Supplementary Figure S1. RNA sample library sizes before and after RLE normalization. Supplementary Figure S2. Distribution of the log2 transformed number of mapped reads per annotated gene for the 40 libraries. Supplementary Figure S3. Kendall’s τ correlation coefficients between pairs of biological replicates (whole transcriptome) (Pdf 321KB).

FILE S3 Supplementary Figures S4–S6. Supplementary Figure S4. Scatter plot showing the relationship of averaged FPKM between pairs of genotypes. Supplementary Figure S5. Pattern of transcriptional activity on core chromosomes. Supplementary Figure S6. Average gene density and transcriptional activity on chromosome 7 (Pdf 671KB).

FILE S4 Supplementary Figure S7. Correlation of fold change of gene expression among selection regimes between genetic backgrounds (Pdf 125KB).

FILE S5 Supplementary Figures S8, S9. Supplementary Figure S8. Distribution of Log2 fold change of all significant genes differentially expressed due to the selection regimes in the model DESeq2*(3)*. Supplementary Figure S9. Volcano plots of DESeq2 analysis model *(3)* for DEGs under fluctuating regime compared to ancestors (Pdf 247KB).

FILE S6 Supplementary Table S2. List of DEGs under fluctuation with repeated evolution (Xlsx 2.5MB).

FILE S7 Supplementary Table S3. Number of DEGs per chromosome for each selection regime and genetic background (Xlsx 13KB).

FILE S8 Supplementary Tables S4, S5. Supplementary Table S4. WGCNA modules (*N* = 16) identified for the background MGGP01. Supplementary Table S5. WGCNA modules (*N* = 15) identified for the background MGGP44 (Pdf 20KB).

FILE S9 Supplementary Figures S10, S11. Supplementary Figure S10. Correlation to the eigengene for the top 8 WGCNA modules according to selection regimes. Supplementary Figure S11. Pairwise average gene connectivity revealed by WGCNA within the 8 selected modules (Docx 6.3MB).

FILE S10 Supplementary Table S6. Description of hub genes. Functional annotation of the 8 hub genes identified using WGCNA approach (Xlsx 13KB).

FILE S11 Supplementary Table S7. Description of the 63 genes which function is associated to transcription regulation or signal transduction, among the top 8 WGCNA modules (Xlsx 19KB).

FILE S12 Supplementary Table S8. Small scale mutations in transcripts common between the two temperatures conditions compared to the reference genome IPO-323 (Xlsx 9.6MB).

FILE S13 Supplementary Table S9. Gene counts according to their change of plasticity among the selection regimes (Xlsx 13KB).

## Abbreviations

AC: accessory chromosome
CC: core chromosome
DEG: differentially expressed gene
FDR: false discovery rate
LRT: likelihood ratio test
PCA: principal component analysis
PDB: potato dextrose broth
QTL: quantitative trait locus
SNP: single nucleotide polymorphism
TE: transposable element
WGCNA: weighted correlation network analysis.

## References

[B1] Benjamini Y. Hochberg Y. (2005). Controlling the false discovery rate: a practical and powerful approach to multiple testing. *J. R. Statist. Soc. B.* 57 289–300. 10.1111/j.2517-6161.1995.tb02031.x

[B2] Bennett A. F. Lenski R. E. Mittler J. E. (1992). Evolutionary adaptation to temperature. I. Fitness responses of Escherichia coli to changes in its thermal environment. *Evolution* 46 16–30. 10.1111/j.1558-5646 28564952

[B3] Bolger A. M. Lohse M. Usadel B. (2014). Trimmomatic: a flexible trimmer for illumina sequence data. *Bioinformatics* 30 2114–2120. 10.1093/bioinformatics/btu170 24695404 PMC4103590

[B4] Brem R. B. Kruglyak L. (2002). Genetic dissection of transcriptional regulation in budding yeast. *Science* 296 752–755. 10.1126/science.1069516 11923494

[B5] Britten R. J. Davidson E. H. (1971). Repetitive and non-repetitive DNA sequences and a speculation on the origins of evolutionary novelty. *Q. Rev. Biol.* 46 111–138. 10.1086/406830 5160087

[B6] Chen J. Nolte V. Schlötterer C. (2015). Temperature-related reaction norms of gene expression: regulatory architecture and functional implications. *Mol. Biol. Evol.* 32 2393–2402. 10.1093/molbev/msv120 25976350 PMC4540970

[B7] Corl A. Bi K. Luke C. Challa A. S. Stern A. J. Sinervo B. (2018). The genetic basis of adaptation following plastic changes in coloration in a novel environment. *Curr. Biol.* 28 2970–2977. 10.1016/j.cub.2018.06.075 30197088

[B8] Croll D. McDonald B. A. (2012). The accessory genome as a cradle for adaptive evolution in pathogens. *PLoS Patho.* 8:e1002608. 10.1371/journal.ppat.1002608 22570606 PMC3343108

[B9] Cuypers T. D. Rutten J. P. Hogeweg P. (2017). Evolution of evolvability and phenotypic plasticity in virtual cells. *BMC Evol. Biol.* 17:60. 10.1186/s12862-017-0918-y 28241744 PMC5329926

[B10] DePristo M. Banks E. Poplin R. Garimella K. Maguire J. Hartl C. (2011). A framework for variation discovery and genotyping using next-generation DNA sequencing data. *Nat. Genet.* 43 491–498. 10.1038/ng.806 21478889 PMC3083463

[B11] Dhar R. Sägesser R. Weikert C. Wagner A. (2013). Yeast adapts to a changing stressful environment by evolving cross-protection and anticipatory gene regulation. *Mol. Biol. Evol.* 30 573–588. 10.1093/molbev/mss253 23125229

[B12] Dong S. M. Raffaele S. Kamoun S. (2015). The two-speed genomes of filamentous pathogens: waltz with plants. *Curr. Opin. Genet. Dev.* 35 57–65. 10.1016/j.gde.2015.09.001 26451981

[B13] Enard W. Khaitovich P. Klose J. Zollner S. Heissig F. (2002). Intra- and interspecific variation in primate gene expression patterns. *Science* 296 340–343. 10.1126/science.1068996 11951044

[B14] Fay J. C. McCullough H. L. Sniegowski P. D. Eisen M. B. (2004). Population genetic variation in gene expression is associated with phenotypic variation in Saccharomyces cerevisiae. *Genome Biol.* 5:R26. 10.1186/gb-2004-5-4-r26 15059259 PMC395785

[B15] Feugeas J. P. Tourret J. Launay A. Bouvet O. Hoede C. Denamur E. (2016). Links between transcription, environmental adaptation and gene variability in *Escherichia coli*: correlations between gene expression and gene variability reflect growth efficiencies. *Mol. Biol. Evol.* 33 2515–2529. 10.1093/molbev/msw105 27352853

[B16] Fischer E. M. Knutti R. (2015). Anthropogenic contribution to global occurrence of heavy-precipitation and high-temperature extremes. *Nat. Clim. Chang.* 46 560–564. 10.1038/NCLIMATE2617

[B17] Fisher K. J. Lang G. I. (2016). Experimental evolution in fungi: an untapped resource. *Fungal Genet. Biol.* 94 88–94. 10.1016/j.fgb.2016.06.007 27375178

[B18] Fones H. Gurr S. (2015). The impact of Septoria tritici Blotch disease on wheat: an EU perspective. *Fungal Genet Biol.* 79 3–7. 10.1016/j.fgb.2015.04.004 26092782 PMC4502551

[B19] Fox R. J. Donelson J. M. Schunter C. Ravasi T. Gaita ìn-Espitia J. D. (2019). Beyond buying time: the role of plasticity in phenotypic adaptation to rapid environmental change. *Phil. Trans. R. Soc. B* 374 20180174. 10.1098/rstb.2018.0174 30966962 PMC6365870

[B20] Francisco C. S. Ma X. Zwyssig M. M. McDonald B. A. Palma-Guerrero J. (2019). Morphological changes in response to environmental stresses in the fungal plant pathogen *Zymoseptoria tritici*. *Sci. Rep.* 9:9642. 10.1038/s41598-019-45994-3 PMC6610121 31270361

[B21] Frantzeskakis L. Kusch S. Panstruga R. (2019). The need for speed: compartmentalized genome evolution in filamentous phytopathogens. *Mol. Plant Pathol.* 20 3–7. 10.1111/mpp.12738 30557450 PMC6430476

[B22] Garland T. Rose M. R. (2009). *Experimental Evolution: Concepts, Methods, and Applications of Selection Experiments.* Berkeley, CL: University of California Press.

[B23] Ghalambor C. K. Hoke K. L. Ruell E. W. Fischer E. K. Reznick D. N. Hughes K. A. (2015). Non-adaptive plasticity potentiates rapid adaptive evolution of gene expression in nature. *Nature* 525 372–375. 10.1038/nature15256 26331546

[B24] Ghalambor C. K. McKay J. K. Carroll S. P. Reznick D. N. (2007). Adaptive versus non-adaptive phenotypic plasticity and the potential for contemporary adaptation in new environments. *Funct. Ecol.* 21 394–407. 10.1111/j.1365-2435.2007.01283.x

[B25] Glaser-Schmitt A. Parsch J. (2018). Functional characterization of adaptive variation within a cis-regulatory element influencing *Drosophila melanogaster* growth. *PLoS Biol.* 16:1–28. 10.1371/journal.pbio.2004538 29324742 PMC5783415

[B26] Goodwin S. B. M’Barek S. Dhillon B. Wittenberg A. H. J. Crane C. F. Hane J. K. (2011). Finished genome of the fungal wheat pathogen *Mycosphaerella graminicola* reveals dispensome structure, chromosome plasticity, and stealth pathogenesis. *PLoS Genet.* 7:e1002070. 10.1371/journal.pgen.1002070 21695235 PMC3111534

[B27] Grandaubert J. Bhattacharyya A. Stukenbrock E. H. (2015). RNA-seq-based gene annotation and comparative genomics of four fungal grass pathogens in the genus Zymoseptoria identify novel orphan genes and species-specific invasions of transposable elements. *G*3 5 1323–1333. 10.1534/g3.115.017731 25917918 PMC4502367

[B28] Hartmann F. E. McDonald B. A. Croll D. (2018). Genome-wide evidence for divergent selection between populations of a major agricultural pathogen. *Mol. Ecol.* 27 2725–2741. 10.1111/mec.14711 29729657 PMC6032900

[B29] Hartmann F. E. Sánchez-Vallet A. McDonald B. A. Croll D. (2017). A fungal wheat pathogen evolved host specialization by extensive chromosomal rearrangements. *ISME J.* 11 1189–1204. 10.1038/ismej.2016.196 28117833 PMC5437930

[B30] Haueisen J. Möller M. Eschenbrenner C. J. Grandaubert J. Seybold H. Adamiak H. (2019). Highly flexible infection programs in a specialized wheat pathogen. *Ecol. Evol.* 9 275–294. 10.1002/ece3.4724 30680113 PMC6342133

[B31] He F. Arce A. L. Schmitz G. Koornneef M. Novikova P. Beyer A. (2016). The footprint of polygenic adaptation on stress-responsive cis-regulatory divergence in the Arabidopsis Genus. *Mol. Biol. Evol.* 33 2088–2101. 10.1093/molbev/msw096 27189540

[B32] Ho W.-C. Zhang J. (2018). Evolutionary adaptations to new environments generally reverse plastic phenotypic changes. *Nat. Comm.* 9:350. 10.1038/s41467-017-02724-5 29367589 PMC5783951

[B33] Huang W. Carbone M. A. Magwire M. M. Peiffer J. A. Lyman R. F. Stone E. A. (2015). Genetic basis of transcriptome diversity in *Drosophila melanogaster*. *PNAS* 112 E6010–E6019. 10.1073/pnas.1519159112 26483487 PMC4640795

[B34] Huang Y. Agrawal A. F. (2016). Experimental evolution of gene expression and plasticity in alternative selective regimes. *PLoS Genet.* 12:e1006336. 10.1371/journal.pgen.1006336 27661078 PMC5035091

[B35] Hughes B. S. Cullum A. J. Bennett A. F. (2007). An experimental evolutionary study on adaptation to temporally fluctuating pH in *Escherichia coli*. *Physiol. Biochem. Zool.* 80 406–421. 10.1086/518353 17508336

[B36] Jallet A. J. Le Rouzic A. And Genissel A. (2019). Evolution and plasticity of the transcriptome under temperature fluctuations in the fungal plant pathogen *Zymoseptoria tritici*. *bioRxiv* 725010. 10.3389/fmicb.2020.573829 PMC7517895 33042084

[B37] Jovic K. Sterken M. G. Grilli J. Bevers R. P. J. Rodriguez M. Riksen J. A. G. (2017). Temporal dynamics of gene expression in heat-stressed *Caenorhabditis elegans*. *PLoS ONE* 12:e0189445. 10.1371/journal.pone.0189445 29228038 PMC5724892

[B38] Kellner R. Bhattacharyya A. Poppe S. Hsu T. Y. Brem R. B. Stukenbrock E. H. (2014). Expression profiling of the wheat pathogen *Zymoseptoria tritici* reveals genomic patterns of transcription and host-specific regulatory programs. *Genome Biol. Evol.* 6 1353–1365. 10.1093/gbe/evu101 24920004 PMC4079195

[B39] Kellogg D. R. (2003). Wee1-dependent mechanisms required for coordination of cell growth and cell division. *J. Cell Sci.* 116 4883–4890. 10.1242/jcs.00908 14625382

[B40] Kenkel C. D. Matz M. V. (2016). Gene expression plasticity as a mechanism of coral adaptation to a variable environment. *Nat. Ecol. Evol.* 7:14. 10.1038/s41559-016-0014 28812568

[B41] King M.-C. Wilson A. C. (1975). Evolution at two levels in humans and chimpanzees. *Science* 188 107–116. 10.1126/science.1090005 1090005

[B42] Lachowiec J. Mason G. A. Schultz K. Queitsch C. (2018). Redundancy, feedback, and robustness in the *Arabidopsis thaliana* BZR/BEH gene family. *Front. Genet.* 9:3389. 10.3389/fgene.2018.00523 30542366 PMC6277886

[B43] Lande R. (2009). Adaptation to an extraordinary environment by evolution of phenotypic plasticity and assimilation. *J. Evol. Biol.* 22 1435–1446. 10.1111/j.1420-9101.2009.01754.x 19467134

[B44] Landry C. R. Oh J. Hartl D. L. Cavalieri D. (2006). Genome-wide scan reveals that genetic variation for transcriptional plasticity in yeast is biased towards multi-copy and dispensable genes. *Gene* 366 343–351. 10.1016/j.gene.2005.10.042 16427747

[B45] Langfelder P. Horvath S. (2008). WGCNA: An R package for weighted correlation network analysis. *BMC Bioinform.* 9:559. 10.1186/1471-2105-9-559 19114008 PMC2631488

[B46] Le Rouzic A. Álvarez-Castro J. M. Hansen T. F. (2013). The evolution of canalization and evolvability in stable and fluctuating environments. *Evol. Biol.* 40 317–340. 10.1007/s11692-012-9218-z

[B47] Le Rouzic A. Carlborg Ö (2008). Evolutionary potential of hidden genetic variation. *Trends Ecol. Evol.* 23 33-37. 10.1016/j.tree.2007.09.014 18079017

[B48] Leder E. H. McCairns R. J. S. Leinonen T. Cano J. M. Viitaniemi H. M. (2015). The evolution and adaptive potential of transcriptional variation in sticklebacks–signatures of selection and widespread heritability. *Mol. Biol. Evol.* 32 674–689. 10.1093/molbev/msu328 25429004 PMC4327155

[B49] Lendenmann M. H. Croll D. Palma-Guerrero J. Stewart E. L. Mcdonald B. A. (2016). QTL mapping of temperature sensitivity reveals candidate genes for thermal adaptation and growth morphology in the plant pathogenic fungus *Zymoseptoria tritici*. *Heredity* 116 384–394. 10.1038/hdy.2015.111 26758189 PMC4806695

[B50] Leroi A. M. Lensky R. E. Bennett A. (1994). Evolutionary adaptation to temperature. III. Adaptation of *Escherichia coli* to a temporally varying environment. *Evolution.* 48 1222–1229. 28564463 10.1111/j.1558-5646.1994.tb05307.x

[B51] Levis N. A. Pfennig D. W. (2016). Evaluating “plasticity-first” evolution in nature:key criteria and empirical approaches. *Trends Ecol. Evol.* 31 563–574. 10.1016/j.tree.2016.03.012 27067134

[B52] Li J. Yuan Z. Zhang Z. (2010). The cellular robustness by genetic redundancy in budding yeast. *PLoS Genet.* 6:e1001187. 10.1371/journal.pgen.1001187 21079672 PMC2973813

[B53] Li Y. Álvarez O. A. Gutteling E. W. Tijsterman M. Fu J. Riksen J. A. G. (2006). Mapping determinants of gene expression plasticity by genetical genomics in *C. elegans*. *PLoS Genet.* 2:2155–2161. 10.1371/journal.pgen.0020222 17196041 PMC1756913

[B54] Love M. I. Huber W. Anders S. (2014). Moderated estimation of fold change and dispersion for RNA-seq data with DESeq2. *Genome Biol.* 15:550. 10.1186/s13059-014-0550-8 25516281 PMC4302049

[B55] McIntyre L. M. (2011). RNA-seq:technical variability and sampling. *BMC Genomics* 12:293. 10.1186/1471-2164-12-293 21645359 PMC3141664

[B56] Meile L. Croll D. Brunner P. C. Plissoneau C. Hartmann F. E. McDonald B. A. (2018). A fungal avirulence factor encoded in a highly plastic genomic region triggers partial resistance to septoria tritici blotch. *New Phytol.* 219 1048–1061. 10.1111/nph.15180 29693722 PMC6055703

[B57] Möller M. Habig M. Freitag M. Stukenbrock E. H. (2018). Extraordinary genome instability and widespread chromosome rearrangements during vegetative growth. *Genetics* 210 517–529. 10.1534/genetics.118.301050 30072376 PMC6216587

[B58] Noormohammad A. Rambeaum J. Held T. Kovakova V. Berg J. Lassig M. (2017). Adaptive evolution of gene expression in Drosophila. *Cell Rep.* 20 1385–1395. 10.1016/j.celrep.2017.07.033 28793262

[B59] O’Driscoll A. O. Kildea S. Doohan F. Spink J. Mullins E. (2014). The wheat – Septoria conflict: a new front opening up? *Trends Plant Sci.* 19 602–610. 10.1016/j.tplants.2014.04.011 24957882

[B60] Oleksiak M. F. Churchill G. A. Crawford D. L. (2002). Variation in gene expression within and among natural populations. *Nat. Genet.* 32 261–266. 10.1038/ng983 12219088

[B61] Osada N. Miyagi R. Takahashi A. (2017). Cis- and trans-regulatory effects on gene expression. *Genetics* 206 2139–2148. 10.1534/genetics.117.201459 28615283 PMC5560811

[B62] Palma-Guerrero J. Ma X. Torriani S. F. F. Zala M. Francisco C. S. Hartmann F. E. (2017). Comparative transcriptome analyses in *Zymoseptoria tritici* reveal significant differences in gene expression among strains during plant infection. *MPMI.* 30 231–244. 10.1094/MPMI-07-16-0146-R 28121239

[B63] Payne J. L. Wagner A. (2019). The causes of evolvability and their evolution. *Nat. Rev. Genet.* 20 24–38. 10.1038/s41576-018-0069-z 30385867

[B64] Pertea M. Kim D. Pertea G. M. Leek J. T. Salzberg S. L. (2016). Transcript-level expression analysis of RNA-seq experiments with HISAT. StringTie and Ballgown. *Nat Protoc.* 11 1650–1667. 10.1038/nprot.2016.095 27560171 PMC5032908

[B65] Pigliucci M. Murre C. J. Schlichting C. D. (2006). Phenotypic plasticity and evolution by genetic assimilation. *J. Exp. Biol.* 209 2362–2367. 10.1242/jeb.02070 16731812

[B66] Podrabsky J. E. (2004). Changes in gene expression associated with acclimation to constant temperatures and fluctuating daily temperatures in an annual killifish Austrofundulus limnaeus. *J. Exp. Biol.* 207 2237–2254. 10.1242/jeb.01016 15159429

[B67] Rockman M. V. Wray G. A. (2002). Abundant raw material for cis-regulatory evolution in humans. *Mol. Biol. Evol.* 19 1991–1994. 10.1093/oxfordjournals.molbev.a004023 12411608

[B68] Rudd J. J. Kanyuka K. Hassani-Pak K. Derbyshire M. Andongabo A. Devonshire J. (2015). Transcriptome and metabolite profiling of the infection cycle of *Zymoseptoria tritici* on wheat reveals a biphasic interaction with plant immunity involving differential pathogen chromosomal contributions and a variation on the hemibiotrophic lifestyle definition. *Plant Physiol.* 167 1158–1185. 10.1104/pp.114.255927 25596183 PMC4348787

[B69] Schadt E. E. Monks S. A. Drake T. A. Lusis A. J. Che N. Colinayo V. (2003). Genetics of gene expression surveyed in maize, mouse and man. *Nature* 422 292–297. 10.1038/nature01434 12646919

[B70] Schotanus K. Soyer J. L. Connolly L. R. Grandaubert J. Happel P. Smith K. M. (2015). Histone modifications rather than the novel regional centromeres of *Zymoseptoria tritici* distinguish core and accessory chromosomes. *Epigenetics Chromatin.* 8:41. 10.1186/s13072-015-0033-5 26430472 PMC4589918

[B71] Schrader L. Schmitz J. (2019). The impact of transposable elements in adaptive evolution. *Mol. Ecol.* 28 1537–1549. 10.1111/mec.14794 30003608

[B72] Siegal M. L. Bergman A. (2002). Waddington’s canalization revisited: Developmental stability and evolution. *PNAS* 99 10528–10532. 10.1073/pnas.102303999 12082173 PMC124963

[B73] Sikkink K. L. Reynolds R. M. Ituarte C. M. Cresko W. A. Phillips P. C. (2019). Environmental and evolutionary drivers of the modular gene regulatory network underlying ophenotypic plasticity for stress resistance in the nematode *Caenorhabditis remanei*. *G*3. 9 969–982. 10.1534/g3.118.200017 30679247 PMC6404610

[B74] Smith E. N. Kruglyak L. (2008). Gene-environment interaction in yeast gene expression. *PLoS Biol.* 6:e83. 10.1371/journal.pbio.0060083 18416601 PMC2292755

[B75] Sørensen J. G. Schou M. F. Kristensen T. N. Loeschcke V. (2016). Thermal fluctuations affect the transcriptome through mechanisms independent of average temperature. *Sci Rep.* 6 30975. 10.1038/srep30975 27487917 PMC4973280

[B76] Steinberg G. (2015). Cell biology of Zymoseptoria tritici: Pathogen cell organization and wheat infection. *Fungal Genet. Biol.* 79 17–23. 10.1016/j.fgb.2015.04.002 26092785 PMC4502449

[B77] Stewart E. L. Croll D. Lendenmann M. H. Sanchez-Vallet A. Hartmann F. E. Palma-Guerrero J. (2018). Quantitative trait locus mapping reveals complex genetic architecture of quantitative virulence in the wheat pathogen *Zymoseptoria tritici*. *Mol Plant Pathol.* 9 201–216. 10.1111/mpp.12515 27868326 PMC6638037

[B78] Whitehead A. Crawford D. L. (2006). Neutral and adaptive variation in gene expression. *PNAS* 103 5425–5430. 10.1073/pnas.0507648103 16567645 PMC1414633

[B79] Wittkopp P. J. Haerum B. K. Clark A. G. (2004). Evolutionary changes in *cis* and *trans* gene regulation. *Nature* 430 85–88. 10.1038/nature02698 15229602

[B80] Young M. D. Wakefield M. J. Smyth G. K. Oshlack A. (2010). Gene ontology analysis for RNA-seq: accounting for selection bias. *Genome Biol.* 11 R14. 10.1186/gb-2010-11-2-r14 20132535 PMC2872874

[B81] Zan Y. Shen X. Forsberg S. K. G. Carlborg Ö (2016). Genetic regulation of transcriptional variation in natural *Arabidopsis thaliana* accessions. *G*3 6 2319–2328. 10.1534/g3.116.030874 27226169 PMC4978887

[B82] Zandveld J. van den Heuvel J. Mulder M. Brakefield P. M. Kirkwood T. B. L. Shanley D. P. (2017). Pervasive gene expression responses to a fluctuating diet in *Drosophila melanogaster*: the importance of measuring multiple traits to decouple potential mediators of life span and reproduction. *Evolution* 71 2572–2583. 10.1111/evo.13327 28833068

[B83] Zhan J. McDonald B. A. (2011). Thermal adaptation in the fungal pathogen *Mycosphaerella graminicola*. *Mol. Ecol.* 20 1689–1701. 10.1111/j.1365-294X.2011.05023.x 21395890

[B84] Zhong Z. Marcel T. C. Hartmann F. E. Ma X. Plissonneau C. Zala M. (2017). A small secreted protein in *Zymoseptoria tritici* is responsible for avirulence on wheat cultivars carrying the Stb6 resistance gene. *New Phytol.* 214 619–631. 10.1111/nph.14434 28164301

[B85] Zhou S. Campbell T. G. Stone E. A. Mackay T. F. C. Anholt R. R. H. (2012). Phenotypic plasticity of the Drosophila transcriptome. *PLoS Genet.* 8:e1002593. 10.1371/journal.pgen.1002593 22479193 PMC3315458

