# Supplementary material for: Evolution and Plasticity of the Transcriptome Under Temperature Fluctuations in the Fungal Plant Pathogen Zymoseptoria tritici
Source: Front Microbiol. 2020 Sep 11;11:573829. doi: 10.3389/fmicb.2020.573829 (PMC7517895; doi:10.3389/fmicb.2020.573829)
Supplement: FILE S1 — Supplementary Table S1. Full list of RNA samples from the experimental evolution used for the differential gene expression analysis (Pdf 94KB). [file Data_Sheet_1.zip › Data Sheet 1.pdf]

Supplementary File 1: Table S1. RNA Samples. Full list of RNA samples from the experimental evolution used for the differential gene expression analysis

No.[1]	Sample IDs	Genetic background	Selection regime	Temperature during selection (°C)	Temperature of assay[2] (°C)	Replicates[3]
1	MGGP01_17_R1	*MGGP01*	-	-	17	R1
	MGGP01_17_R2	*MGGP01*	-	-	17	R2
2	MGGP01_23_R1	*MGGP01*	-	-	23	R1
	MGGP01_23_R2	*MGGP01*	-	-	23	R2
3	MGGP01_23_17_R1	*MGGP01*	Stable	23	17	R1
	MGGP01_23_17_R2	*MGGP01*	Stable	23	17	R2
4	MGGP01_23_23_R1	*MGGP01*	Stable	23	23	R1
	MGGP01_23_23_R2	*MGGP01*	Stable	23	23	R2
5	MGGP01_17_17_R1	*MGGP01*	Stable	17	17	R1
	MGGP01_17_17_R2	*MGGP01*	Stable	17	17	R2
6	MGGP01_17_23_R1	*MGGP01*	Stable	17	23	R1
	MGGP01_17_23_R2	*MGGP01*	Stable	17	23	R2
7	MGGP01_F_1_17_R1	*MGGP01*	Fluctuating	17/23	17	R1
	MGGP01_F_1_17_R2	*MGGP01*	Fluctuating	17/23	17	R2
8	MGGP01_F_1_23_R1	*MGGP01*	Fluctuating	17/23	23	R1
	MGGP01_F_1_23_R2	*MGGP01*	Fluctuating	17/23	23	R2
9	MGGP01_F_2_17_R1	*MGGP01*	Fluctuating	17/23	17	R1
	MGGP01_F_2_17_R2	*MGGP01*	Fluctuating	17/23	17	R2
10	MGGP01_F_2_23_R1	*MGGP01*	Fluctuating	17/23	23	R1
	MGGP01_F_2_23_R2	*MGGP01*	Fluctuating	17/23	23	R2
11	MGGP44_17_R1	*MGGP44*	-	-	17	R1
	MGGP44_17_R2	*MGGP44*	-	-	17	R2
12	MGGP44_23_R1	*MGGP44*	-	-	23	R1
	MGGP44_23_R2	*MGGP44*	-	-	23	R2

13	MGGP44_23_17_R1	*MGGP44*	Stable	23	17	R1
	MGGP44_23_17_R2	*MGGP44*	Stable	23	17	R2
14	MGGP44_23_23_R1	*MGGP44*	Stable	23	23	R1
	MGGP44_23_23_R2	*MGGP44*	Stable	23	23	R2
15	MGGP44_17_17_R1	*MGGP44*	Stable	17	17	R1
	MGGP44_17_17_R2	*MGGP44*	Stable	17	17	R2
16	MGGP44_17_23_R1	*MGGP44*	Stable	17	23	R1
	MGGP44_17_23_R2	*MGGP44*	Stable	17	23	R2
17	MGGP44_F_1_17_R1	*MGGP44*	Fluctuating	17/23	17	R1
	MGGP44_F_1_17_R2	*MGGP44*	Fluctuating	17/23	17	R2
18	MGGP44_F_1_23_R1	*MGGP44*	Fluctuating	17/23	23	R1
	MGGP44_F_1_23_R2	*MGGP44*	Fluctuating	17/23	23	R2
19	MGGP44_F_2_17_R1	*MGGP44*	Fluctuating	17/23	17	R1
	MGGP44_F_2_17_R2	*MGGP44*	Fluctuating	17/23	17	R2
20	MGGP44_F_2_23_R1	*MGGP44*	Fluctuating	17/23	23	R1
	MGGP44_F_2_23_R2	*MGGP44*	Fluctuating	17/23	23	R2

[1] Number assigned to each pair of biological replicates [2] RNA were collected after one week of fungal multiplication at 17°C or 23°C [3] Two independent biological replicates were produced per lineage
