# Supplementary material for: Evolution and Plasticity of the Transcriptome Under Temperature Fluctuations in the Fungal Plant Pathogen Zymoseptoria tritici
Source: Front Microbiol. 2020 Sep 11;11:573829. doi: 10.3389/fmicb.2020.573829 (PMC7517895; doi:10.3389/fmicb.2020.573829)
Supplement: FILE S1 — Supplementary Table S1. Full list of RNA samples from the experimental evolution used for the differential gene expression analysis (Pdf 94KB). [file Data_Sheet_1.zip › Data Sheet 2.pdf]

Supplementary File 2

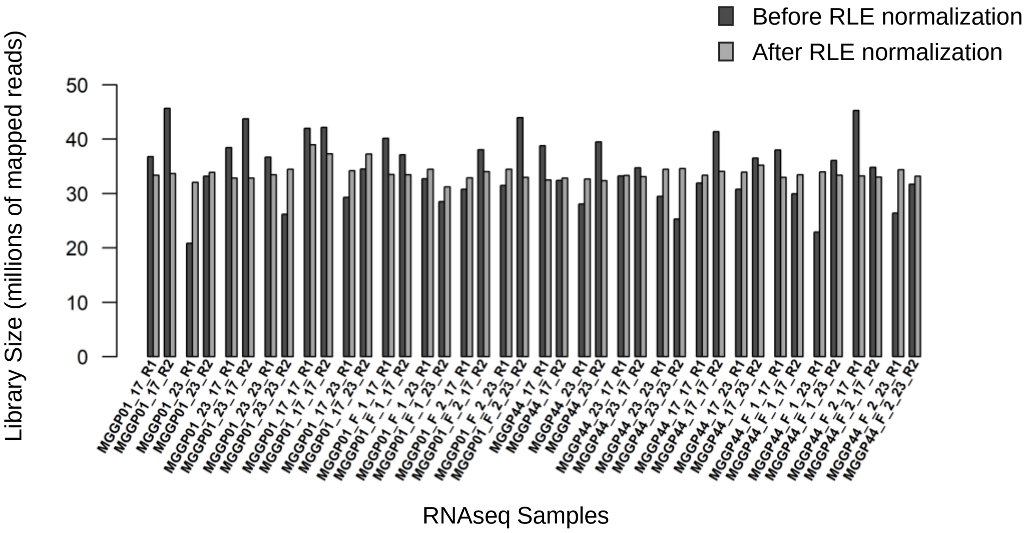

Fig. S1. Variation of RNA sample library size before and after RLE normalization. Sample labels are fully described in Table S1. Two different genetic backgrounds were tested (*MGGP01* and *MGGP44*); 3 different selection regimes were tested (stable 17°C (_17), stable 23°C (_23) and fluctuating temperature between 17°C and 23°C (_F)); 2 experimental replicates for the fluctuating regime were performed (F_1, F_2); 2 temperatures of assay were tested prior to sequencing: 17°C and 23°C (_17, _23); and biological replicates are annotated _R1 and _R2. Reads were mapped using the annotation of the reference genome of *Z. tritici* from (Goodwin et al., 2011).

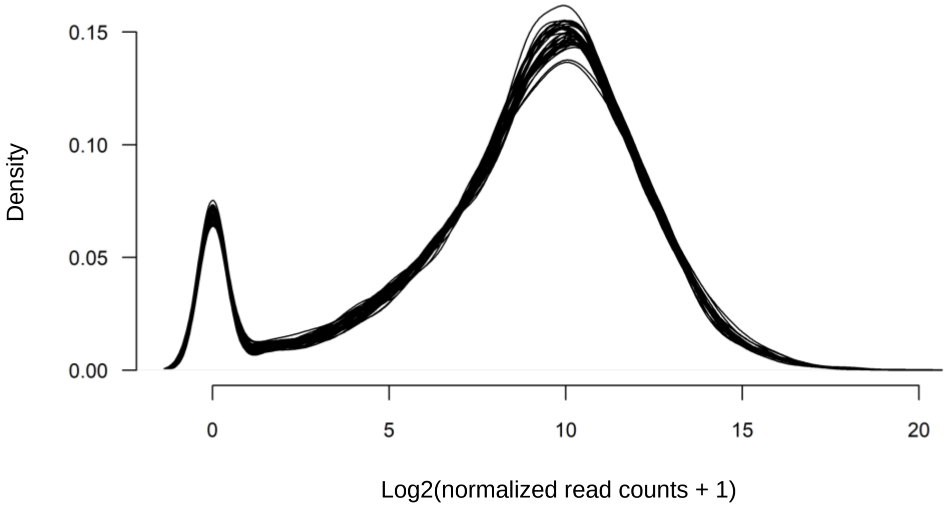

Figure S2. Distribution of the log2 transformed number of mapped reads per annotated gene for the 40 libraries.

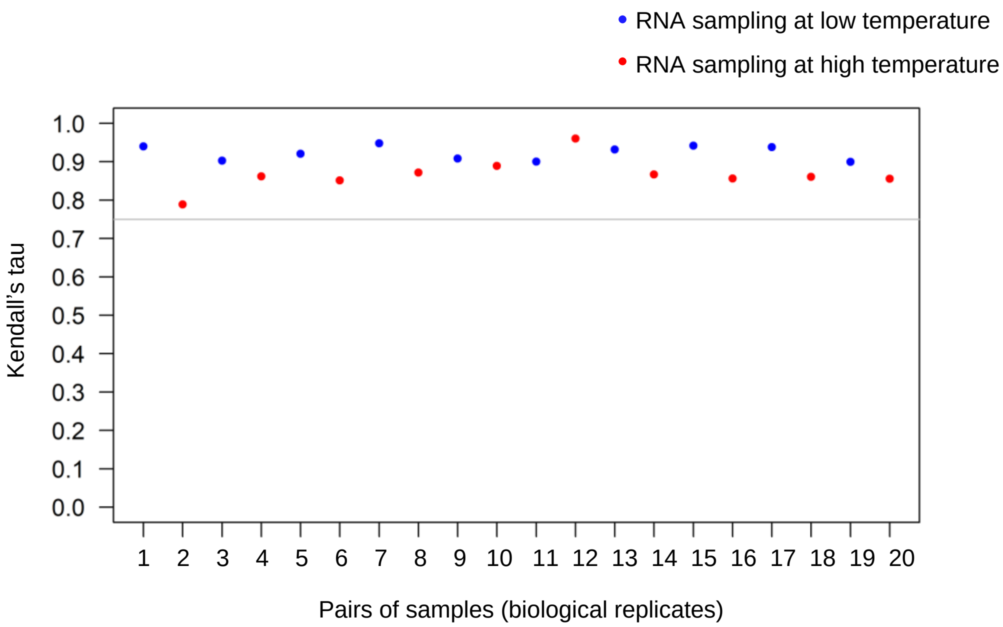

Figure S3. Kendall's τ correlation coefficient between pairs of biological replicates estimated using whole transcriptomes. Corresponding sample names for each pair of samples are listed in Table S1. Grey horizontal line represents the τ value of 0.75.
