# Supplementary material for: Evolution and Plasticity of the Transcriptome Under Temperature Fluctuations in the Fungal Plant Pathogen Zymoseptoria tritici
Source: Front Microbiol. 2020 Sep 11;11:573829. doi: 10.3389/fmicb.2020.573829 (PMC7517895; doi:10.3389/fmicb.2020.573829)
Supplement: FILE S1 — Supplementary Table S1. Full list of RNA samples from the experimental evolution used for the differential gene expression analysis (Pdf 94KB). [file Data_Sheet_1.zip › Data Sheet 3.pdf]

Supplementary File 3

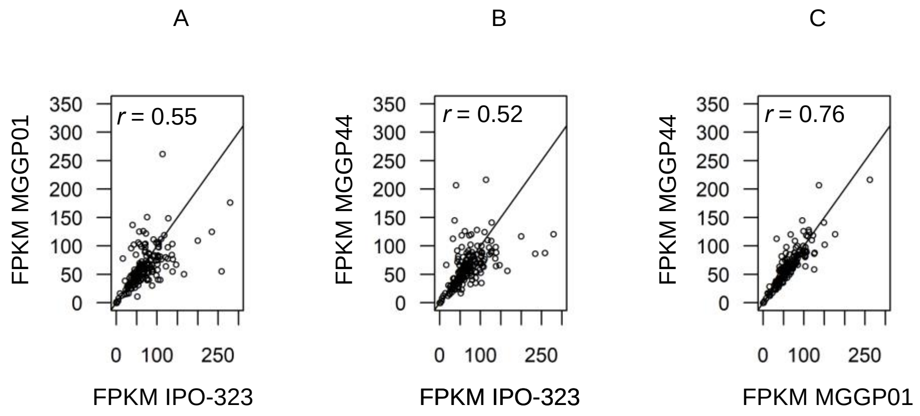

Figure S4. Scatter plot showing the relationship of averaged FPKM between pairs of genotypes. Average FPKM was measured from 181 non-overlapping windows of 200 kb. Pearson's correlation coefficients are indicated. Genotypes IPO-323 *versus* MGGP01 (**A**), genotypes IPO-323 versus MGGP44 (**B**) and genotypes MGGP01 *versus* MGGP44 (**C**). The black line corresponds to the first bisector (y = x).

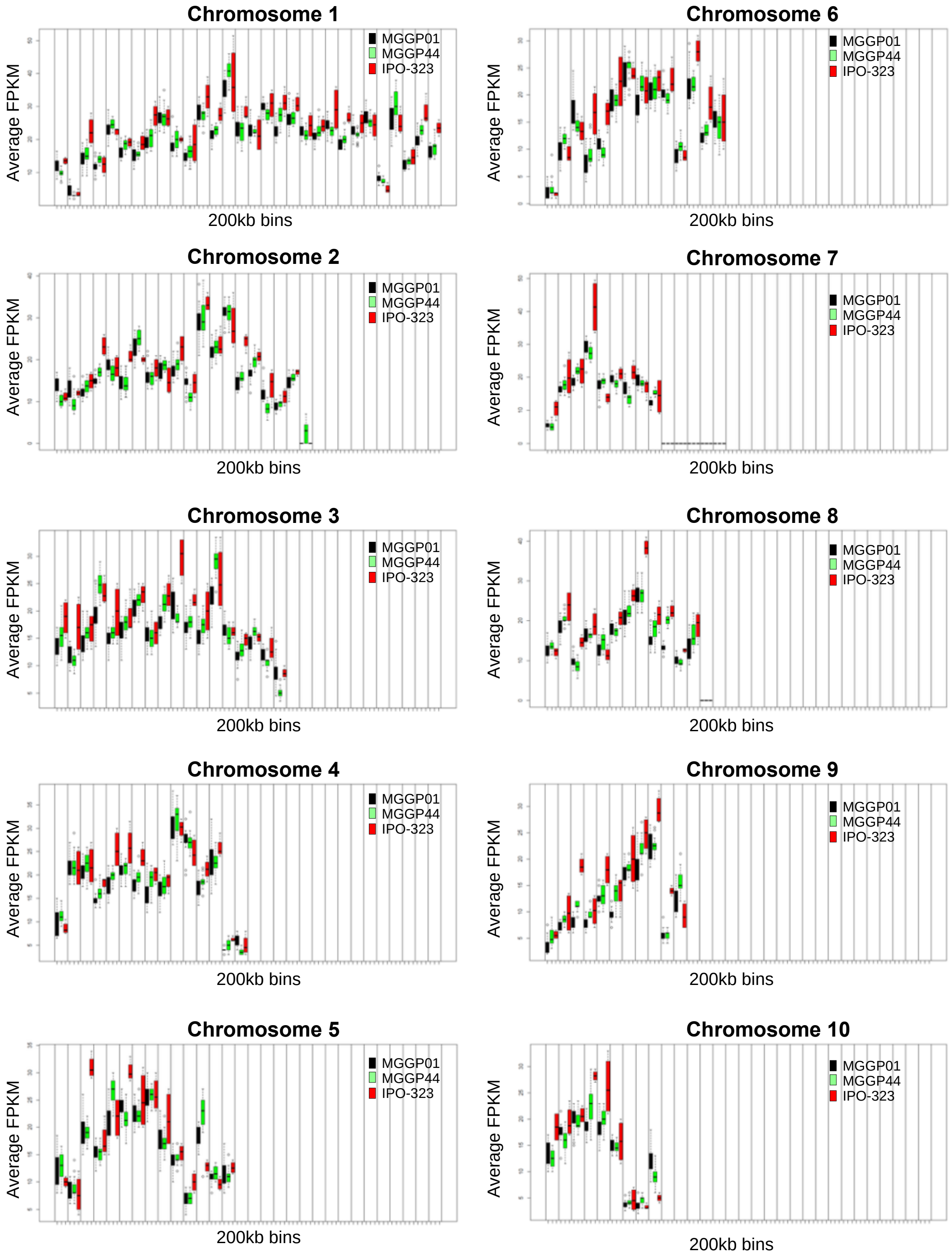
Chromosome 1
MGGP01
MGGP44
IPO-323
Average FPKM
200kb bins
Chromosome 6
MGGP01
MGGP44
IPO-323
Average FPKM
200kb bins
Chromosome 2
MGGP01
MGGP44
IPO-323
Average FPKM
200kb bins
Chromosome 7
MGGP01
MGGP44
IPO-323
Average FPKM
200kb bins
Chromosome 3
MGGP01
MGGP44
IPO-323
Average FPKM
200kb bins
Chromosome 8
MGGP01
MGGP44
IPO-323
Average FPKM
200kb bins
Chromosome 4
MGGP01
MGGP44
IPO-323
Average FPKM
200kb bins
Chromosome 9
MGGP01
MGGP44
IPO-323
Average FPKM
200kb bins
Chromosome 5
MGGP01
MGGP44
IPO-323
Average FPKM
200kb bins
Chromosome 10
MGGP01
MGGP44
IPO-323
Average FPKM
200kb bins

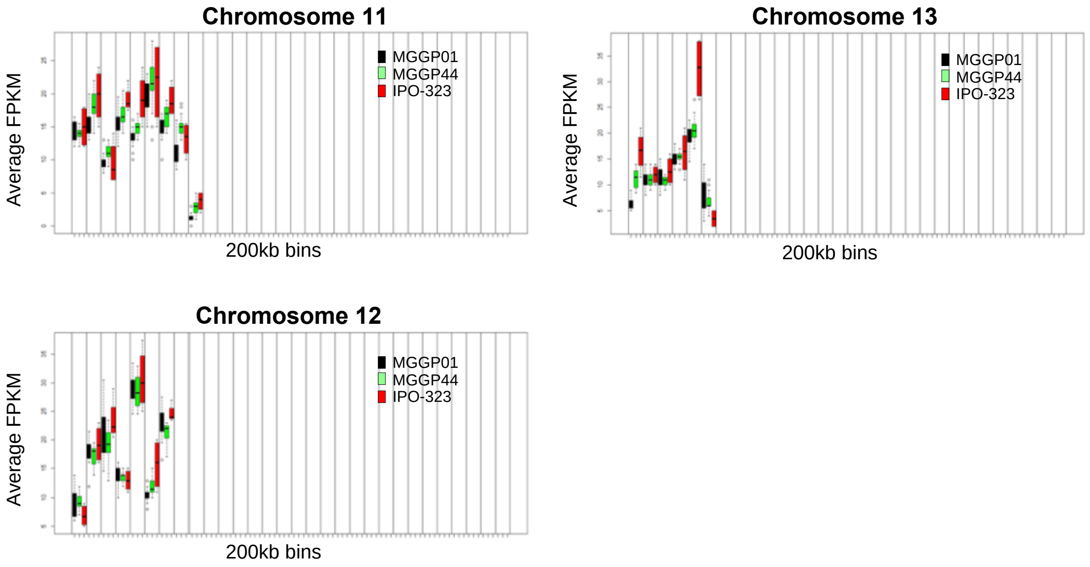

Figure S5. Profile of transcriptional activity on core genome. Average FPKM within 200kb non-overlapping windows along the core chromosomes (1-13).

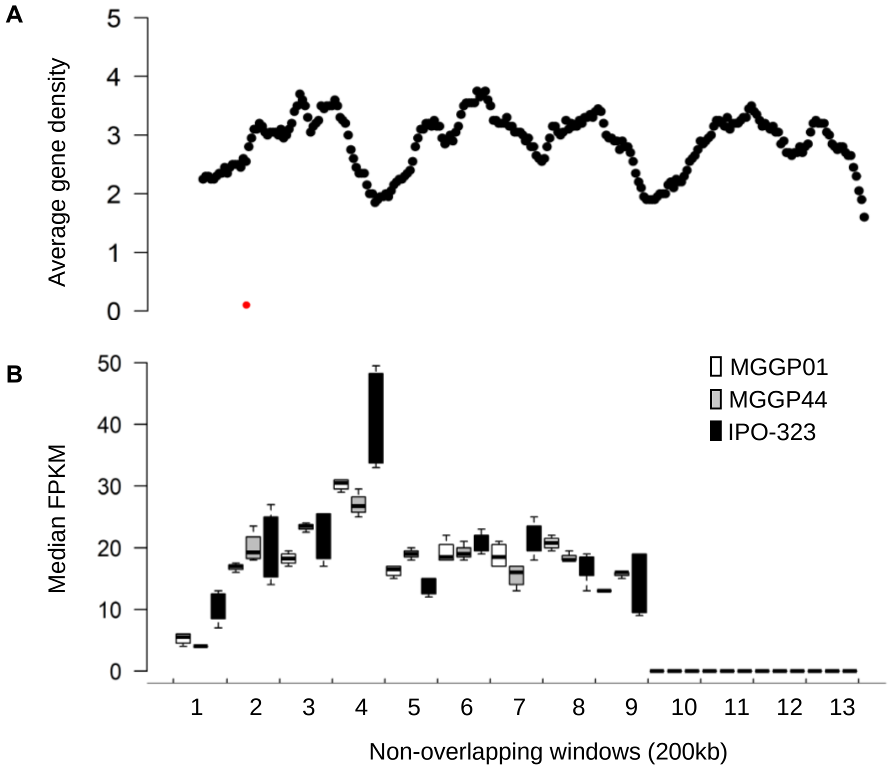

Figure S6. Average gene density and profile of transcriptional activity on chromosome 7. Gene density (30kb overlapping windows by 10kb). Red dot: centromere position (**A**); Gene expression profile for the isolates using median FPKM within 200kb non-overlapping windows along the chromosome 7 (**B**).
