# Supplementary material for: Evolution and Plasticity of the Transcriptome Under Temperature Fluctuations in the Fungal Plant Pathogen Zymoseptoria tritici
Source: Front Microbiol. 2020 Sep 11;11:573829. doi: 10.3389/fmicb.2020.573829 (PMC7517895; doi:10.3389/fmicb.2020.573829)
Supplement: FILE S1 — Supplementary Table S1. Full list of RNA samples from the experimental evolution used for the differential gene expression analysis (Pdf 94KB). [file Data_Sheet_1.zip › Data Sheet 4.pdf]

Supplementary File 4

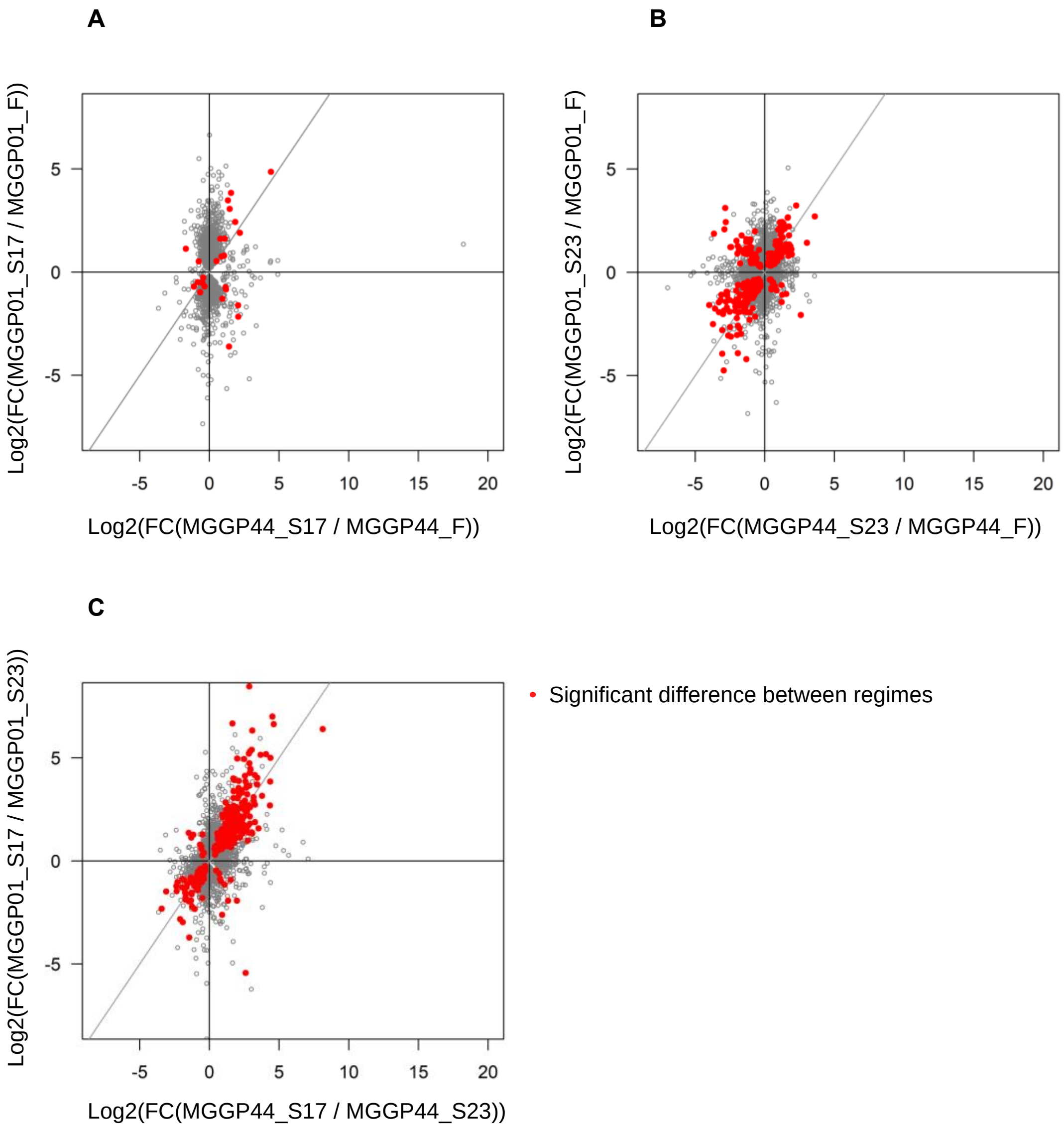

Figure S7. Comparison of fold change of gene expression among selection regimes between genetic backgrounds. All significant genes (1728) for the interaction term Regime-by-Genetic background in model DESeq2 (2) are included; red dots: significant contrasts; grey line: first bissector (y=x) **A:** Fold change of gene expression between Stable at 17°C and Fluctuating lineages, Spearman correlation coefficient $\rho = 0.23$; **B:** Fold change of gene expression between Stable at 23°C and Fluctuating lineages, Spearman correlation coefficient $\rho = 0.31$; **C:** Fold change of gene expression between Stable at 17°C and Stable 23°C lineages, Spearman correlation coefficient $\rho = 0.51$.
