# Supplementary material for: Evolution and Plasticity of the Transcriptome Under Temperature Fluctuations in the Fungal Plant Pathogen Zymoseptoria tritici
Source: Front Microbiol. 2020 Sep 11;11:573829. doi: 10.3389/fmicb.2020.573829 (PMC7517895; doi:10.3389/fmicb.2020.573829)
Supplement: FILE S1 — Supplementary Table S1. Full list of RNA samples from the experimental evolution used for the differential gene expression analysis (Pdf 94KB). [file Data_Sheet_1.zip › Data Sheet 5.pdf]

Supplementary File 5

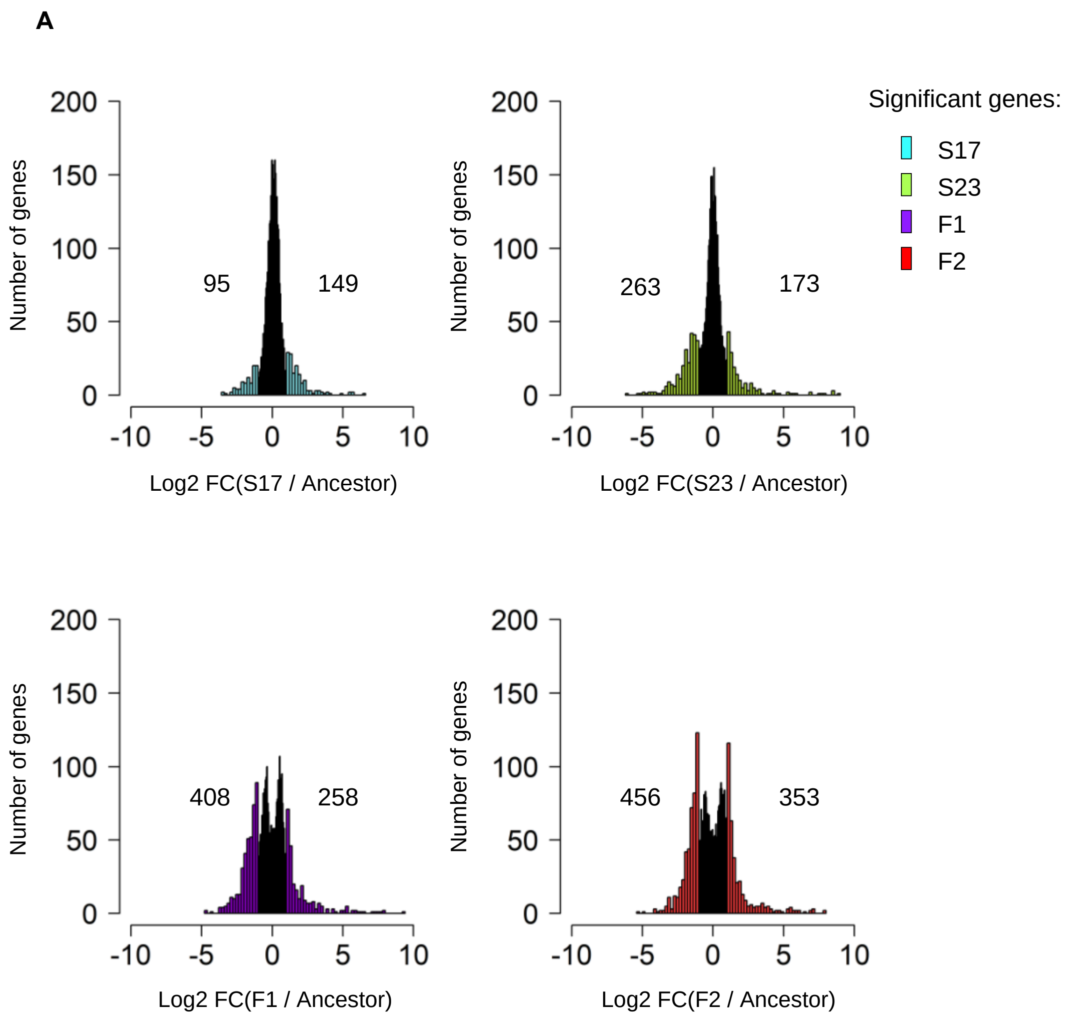

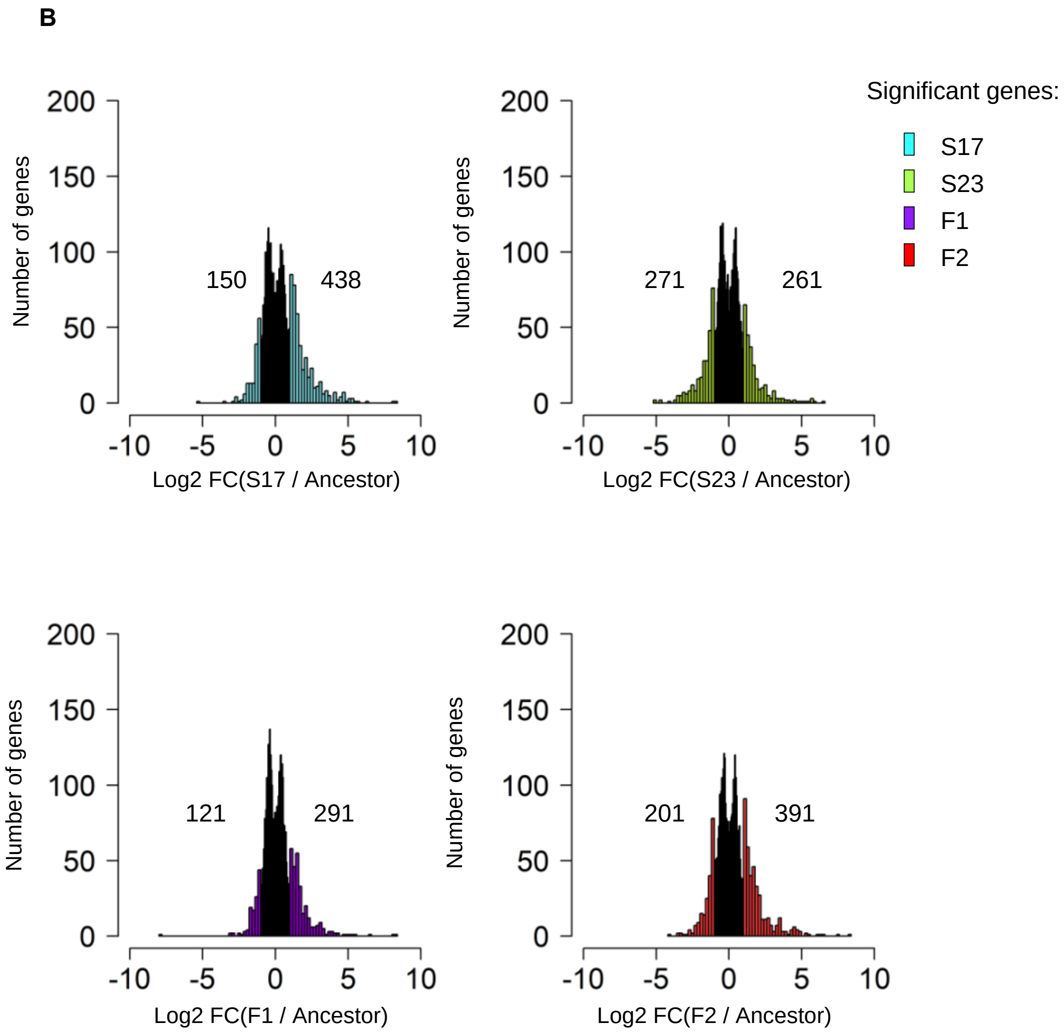

Figure S8. Distribution of Log2 fold change of all significant genes differentially expressed due to the selection regimes in the model DESeq2 *(3)*. A: results for the genetic background MGGP01 (3420 genes) ; **B:** results for the genetic background MGGP44 (3659 genes); number of up- and down-regulated significant genes are indicated on top of each graph.

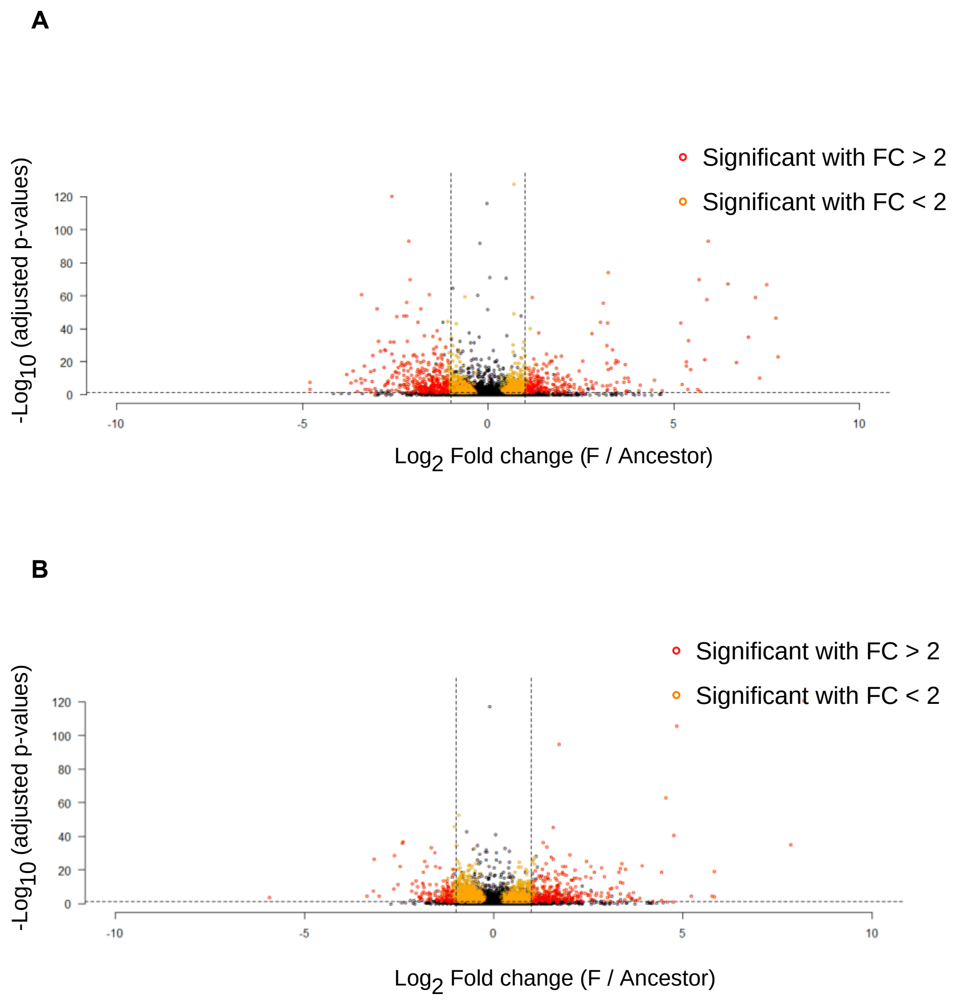

Figure S9. Volcano plots of DESeq2 analysis model *(3)* for DEGs under fluctuating regime compared to ancestors. A: results for the genetic background MGGP01 ; **B:** results for the genetic background MGGP44 ; vertical dashed lines: raw fold change of 2 ; horizontal dashed line: FDR threshold at 5 %.
