# Supplementary material for: Evolution and Plasticity of the Transcriptome Under Temperature Fluctuations in the Fungal Plant Pathogen Zymoseptoria tritici
Source: Front Microbiol. 2020 Sep 11;11:573829. doi: 10.3389/fmicb.2020.573829 (PMC7517895; doi:10.3389/fmicb.2020.573829)
Supplement: FILE S1 — Supplementary Table S1. Full list of RNA samples from the experimental evolution used for the differential gene expression analysis (Pdf 94KB). [file Data_Sheet_1.zip › Data Sheet 8.pdf]

Supplementary File 8

Table S4. WGCNA modules (N=16) identified for the background MGGP01. Each module contains genes which expression profiles across selection regimes are similar.

**WGCNA module**	**Gene count**
MGGP01_blue	760
MGGP01_darkolivegreen	622
MGGP01_black	556
MGGP01_magenta	532
MGGP01_darkgreen	458
MGGP01_skyblue	456
MGGP01_red	356
MGGP01_darkred	227
MGGP01_purple	177
MGGP01_tan	166
MGGP01_orange	154
MGGP01_darkorange	151
MGGP01_lightyellow	125
MGGP01_darkmagenta	90
MGGP01_white	77
MGGP01_yellowgreen	41

Table S5. WGCNA modules (N=15) identified for the background MGGP44. Each module contains genes which expression profiles across selection regimes are similar.

WGCNA module	Gene count
MGGP44_grey60	1509
MGGP44_lightcyan	1455
MGGP44_black	409
MGGP44_salmon	313
MGGP44_green	304
MGGP44_cyan	224
MGGP44_darkolivegreen	185
MGGP44_darkmagenta	181
MGGP44_tan	160
MGGP44_darkorange	144
MGGP44_orange	101
MGGP44_skyblue	93
MGGP44_steelblue	85
MGGP44_violet	76
MGGP44_sienna3	66
