# Supplementary material for: Evolution and Plasticity of the Transcriptome Under Temperature Fluctuations in the Fungal Plant Pathogen Zymoseptoria tritici
Source: Front Microbiol. 2020 Sep 11;11:573829. doi: 10.3389/fmicb.2020.573829 (PMC7517895; doi:10.3389/fmicb.2020.573829)
Supplement: FILE S1 — Supplementary Table S1. Full list of RNA samples from the experimental evolution used for the differential gene expression analysis (Pdf 94KB). [file Data_Sheet_1.zip › Data Sheet 9.pdf]

Supplementary File 9

A

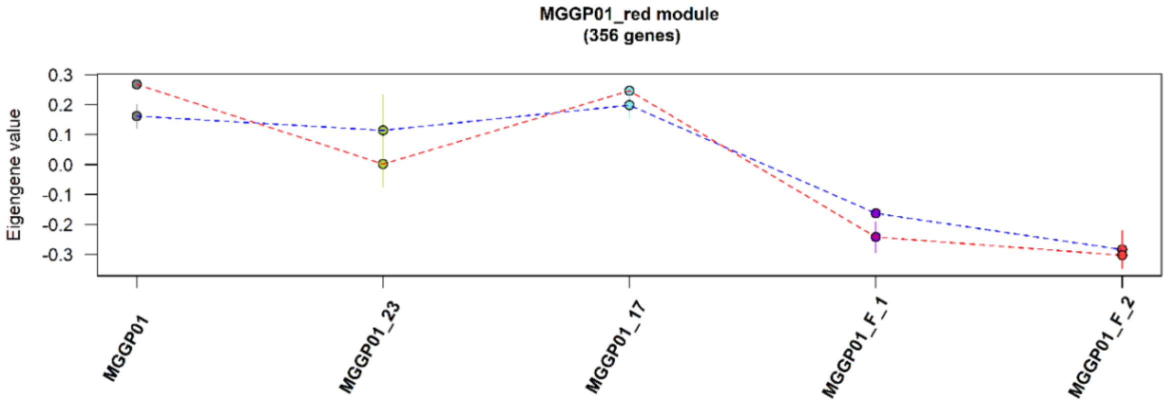

B

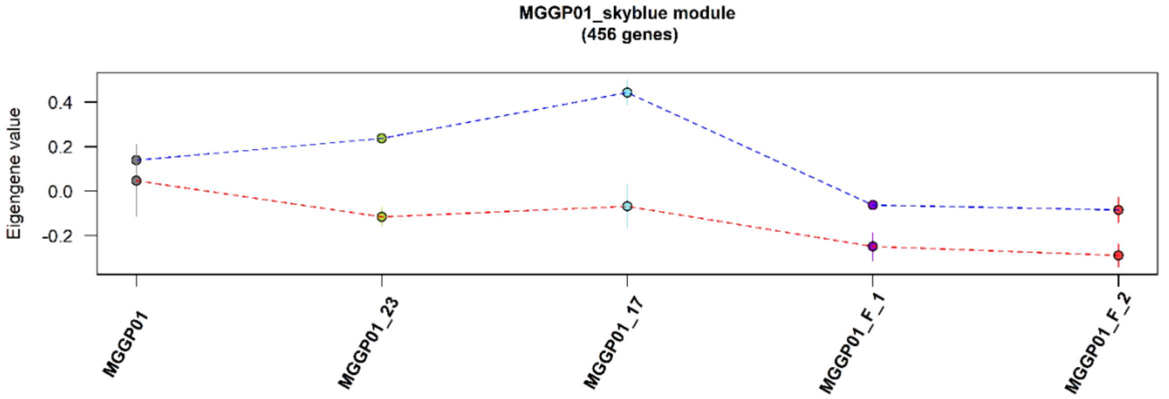

C

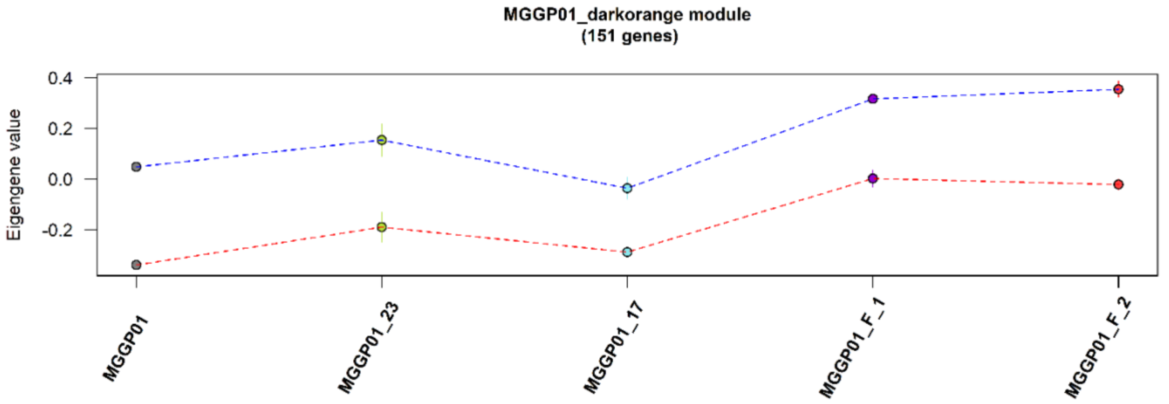

D

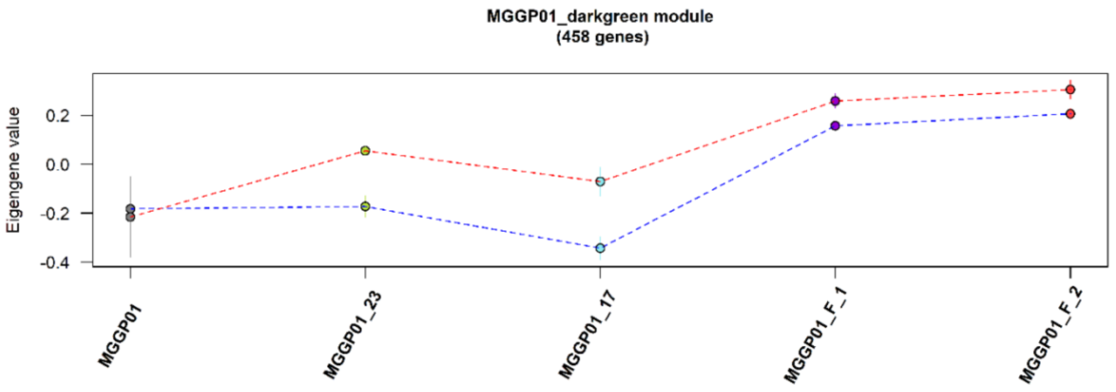
MGGP01_darkgreen module
(458 genes)
Eigengene value
0.2
0.0
-0.2
-0.4
MGGP01
MGGP01_23
MGGP01_17
MGGP01_F_1
MGGP01_F_2

E

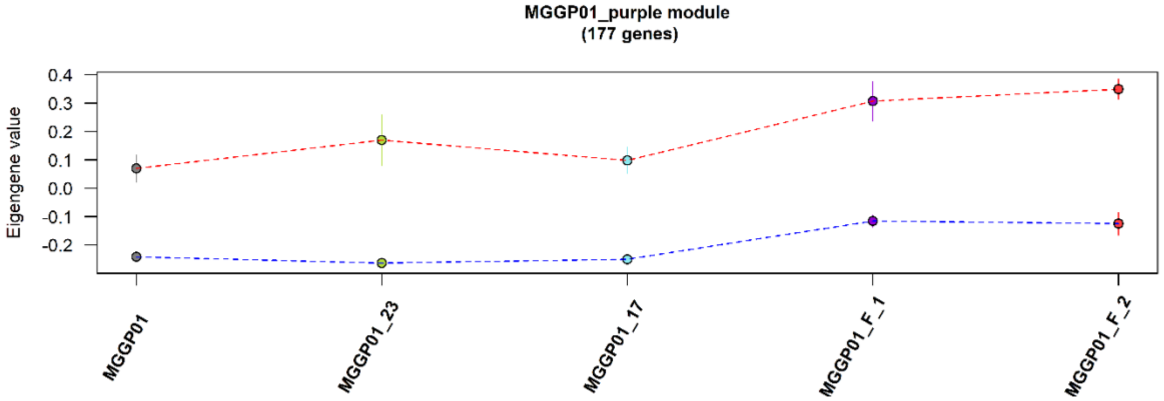
MGGP01_purple module
(177 genes)
Eigengene value
0.4
0.3
0.2
0.1
0.0
-0.1
-0.2
MGGP01
MGGP01_23
MGGP01_17
MGGP01_F_1
MGGP01_F_2

F

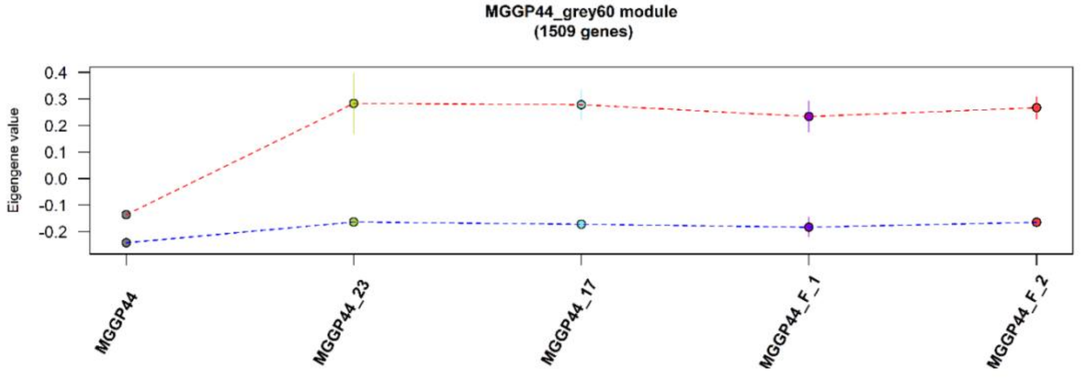
MGGP44_grey60 module
(1509 genes)
Eigengene value
0.4
0.3
0.2
0.1
0.0
-0.1
-0.2
MGGP44
MGGP44_23
MGGP44_17
MGGP44_F_1
MGGP44_F_2

G

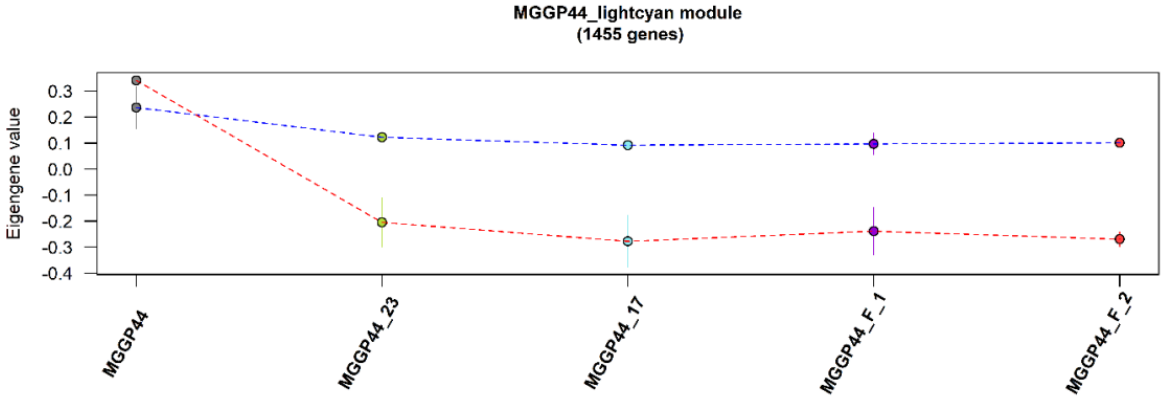

H

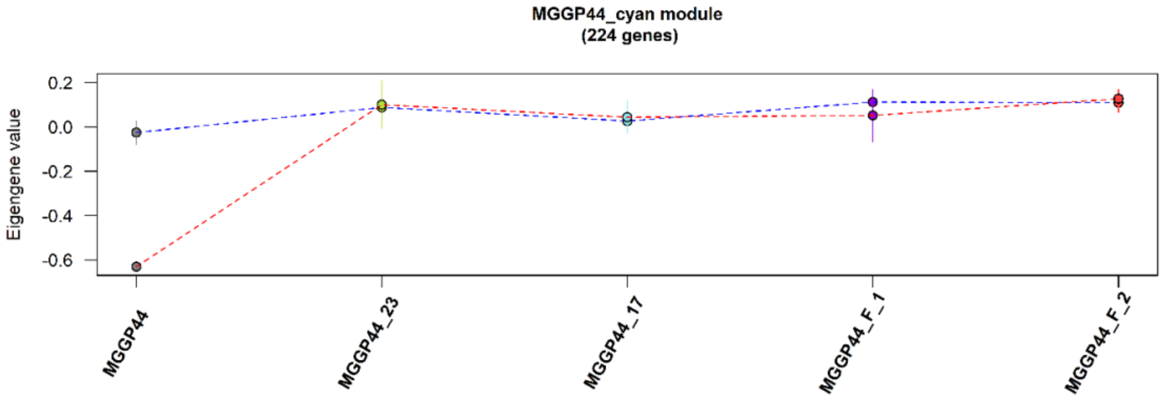

Figure S10. Pattern of the eigengene values among the evolved lineages for the 8 selected WGCNA modules. A to E: Five modules detected for the background MGGP01; **F to H**: Three modules detected for the background MGGP44; blue dashed line: gene expression at 17°C, red dashed line: gene expression at 23°C; Within module eigengene value is given for each evolved lineage is given: stable 23°C, stable 17°C and two fluctuating temperature replicates.

A

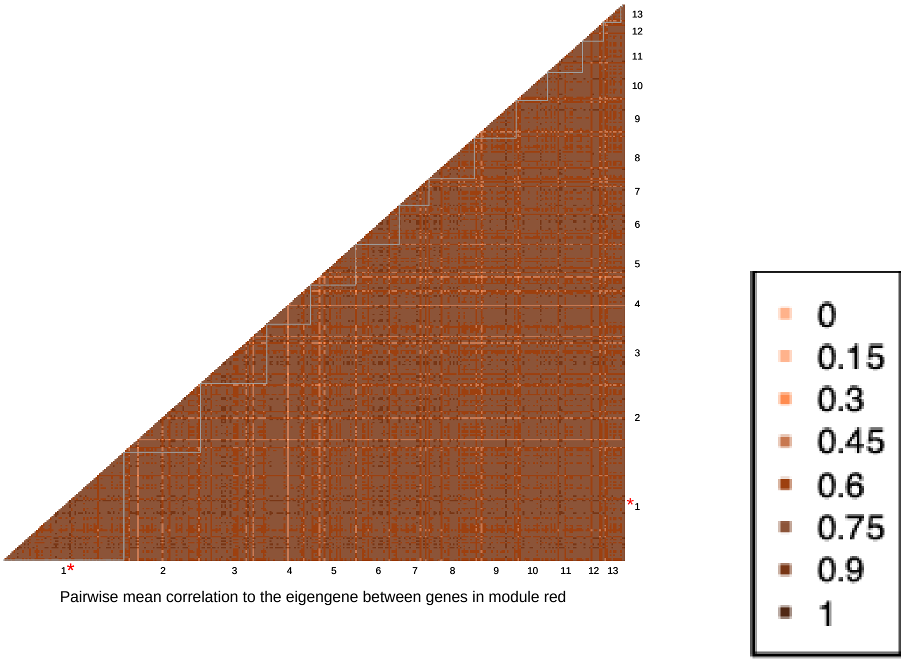

Pairwise mean correlation to the eigengene between genes in module red

B

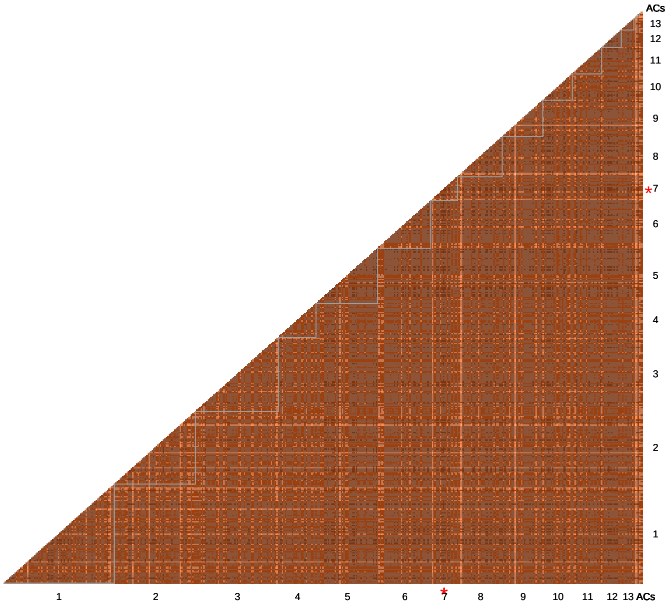

Pairwise mean correlation to the eigengene between genes in module skyblue

C

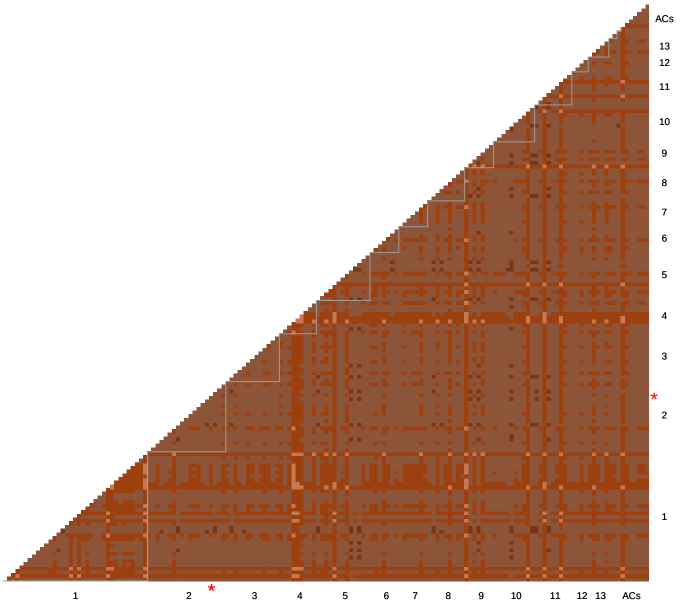

Pairwise mean correlation to the eigengene between genes in module darkorange

D

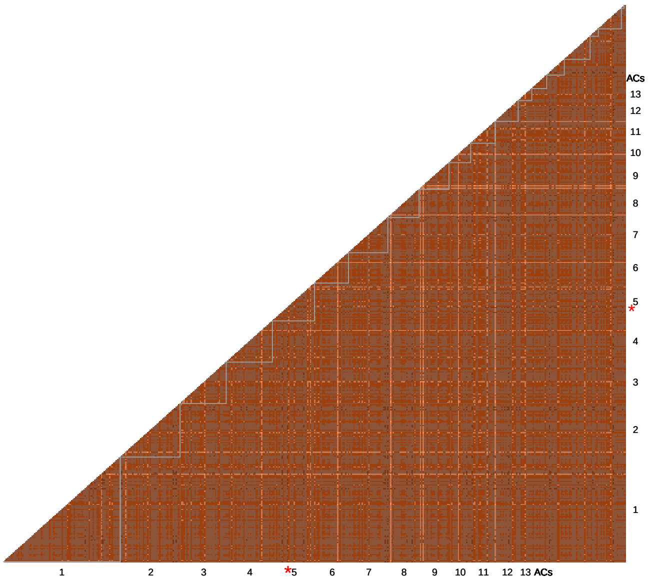

Pairwise mean correlation to the eigengene between genes in module darkgreen

E

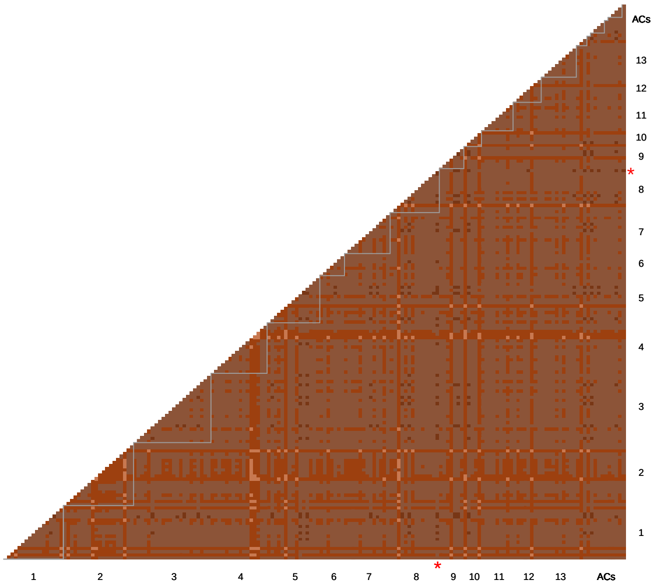

Pairwise mean correlation to the eigengene between genes in module purple

F

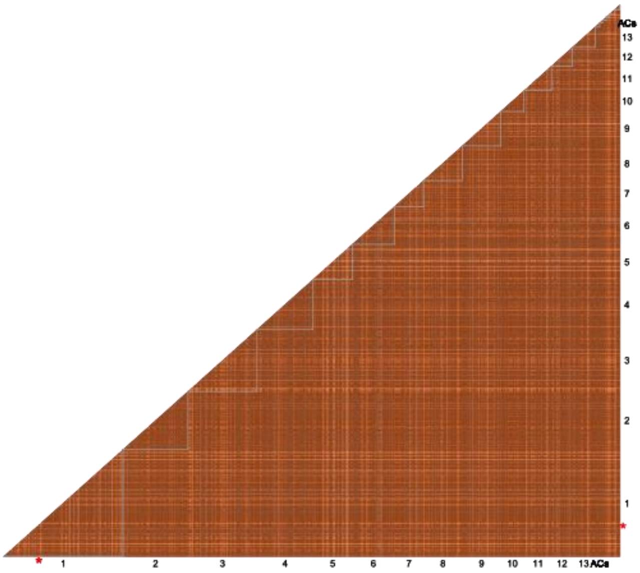

Pairwise mean correlation to the eigengene between genes in module Grey60

G

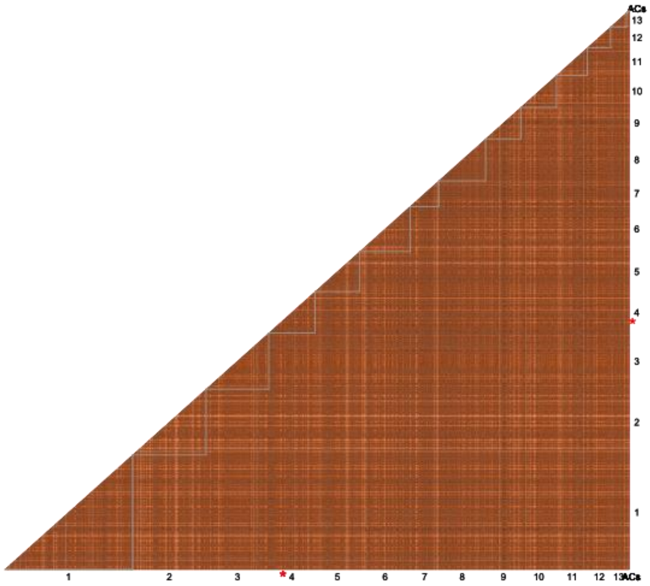

Pairwise mean correlation to the eigengene between genes in module lightcyan

H

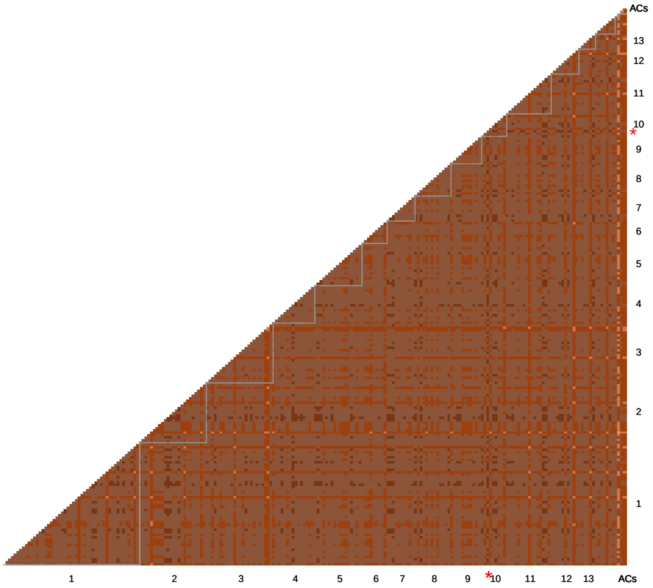

Pairwise mean correlation to the eigengene between genes in module cyan

Figure S11. Pairwise average gene connectivity revealed by WGCNA within the 8 selected modules. Gene connectivity is measured by the correlation of each gene to the eigengene within a module (**A** to **E** : genotype MGGP01; **F** to **H**: genotype MGGP44). Grey lines are delimiting pairwise comparisons within chromosomes; hub gene position is indicated by the red star.
